# ICE1 and ZOU determine the depth of primary seed dormancy in Arabidopsis independently of their role in endosperm development

**DOI:** 10.1111/tpj.14211

**Published:** 2019-02-18

**Authors:** Dana R. MacGregor, Naichao Zhang, Mayumi Iwasaki, Min Chen, Anuja Dave, Luis Lopez‐Molina, Steven Penfield

**Affiliations:** ^1^ Department of Crop Genetics John Innes Centre Norwich Research Park Norwich NR4 7UH UK; ^2^ Department of Plant Biology and Institute for Genetics and Genomics in Geneva (iGE3) University of Geneva 30, Quai Ernest‐Ansermet CH‐1211 Geneva 4 Switzerland; ^3^ Present address: Department of Biointeractions and Crop Protection Rothamsted Research Harpenden, Hertfordshire AL5 2JQ UK; ^4^ Present address: College of Life Science and Technology Huazhong Agricultural University Wuhan 430070 China

**Keywords:** ABA, ABI3, endosperm consumption, ICE1, primary dormancy, seed development, ZOU

## Abstract

Seed dormancy is a widespread and key adaptive trait that is essential for the establishment of soil seed banks and prevention of pre‐harvest sprouting. Herein we demonstrate that the endosperm‐expressed transcription factors *ZHOUPI* (*ZOU*) and *INDUCER OF CBF EXPRESSION1* (*ICE1*) play a role in determining the depth of primary dormancy in Arabidopsis. We show that *ice1* or *zou* increases seed dormancy and the double mutant has an additive phenotype. This increased dormancy is associated with increased ABA levels, and can be separated genetically from any role in endosperm maturation because loss of ABA biosynthesis or *DELAY OF GERMINATION 1* reverses the dormancy phenotype without affecting the aberrant seed morphology. Consistent with these results, *ice1* endosperms had an increased capacity for preventing embryo greening, a phenotype previously associated with an increase in endospermic ABA levels. Although *ice1* changes the expression of many genes, including some in ABA biosynthesis, catabolism and/or signalling, only *ABA INSENSITIVE 3* is significantly misregulated in *ice1* mutants. We also demonstrate that ICE1 binds to and inhibits expression of *ABA INSENSITIVE 3*. Our data demonstrate that Arabidopsis ICE1 and ZOU determine the depth of primary dormancy during maturation independently of their effect on endosperm development.

## Significance Statement

Our data demonstrate that ICE1 and ZOU determine the depth of primary seed dormancy and that this can be separated from their role in initiating endosperm consumption. These genes effect endospermic ABA production and ICE1 binds to and regulates the expression of *ABA INSENSITIVE 3*, one of the *AFL* transcription factors well known for their role in inducing both seed dormancy and the seed maturation programme in the embryo and endosperm.

## Introduction

After fertilisation, seeds enter a rigid developmental programme which proceeds through embryogenesis to seed maturation, where the basic body plan of the plant is established, desiccation tolerance is gained and primary dormancy is imposed (Baud *et al*., 2002; Fourquin *et al*., 2016).The plant hormone abscisic acid (ABA) and a small network of B3‐family transcription factors including *ABA INSENSITIVE 3* (*ABI3*), *FUSCA3* and *LEAFY COTYLEDON 2*, otherwise known as the *AFL* subfamily of B3 transcription factors, induce the seed maturation programme in the embryo and endosperm, as well as seed dormancy (Karssen *et al*., 1983; Koornneef *et al*., 1984; Giraudat *et al*., 1992; Parcy *et al*., 1994; Nambara *et al*., 1995; Lopez‐Molina *et al*., 2002).

Abscisic acid and *ABI3* continue to be important upon seed imbibition when they are required to block the germination of dormant seeds (reviewed in Koornneef *et al*., 2002; Carbonero *et al*., 2017; Leprince *et al*., 2017). After shedding, primary dormancy can be broken by environmental signals such as seasonal changes in temperature or soil nitrate levels or signals of canopy disturbance such as compounds in smoke from forest fires (Finch‐Savage and Leubner‐Metzger, 2006). In the laboratory, these environmental responses are exploited to create simple dormancy‐breaking treatments such as cold stratification or dry after‐ripening which are often used as methods for comparing the depth of seed dormancy between genotypes.

Depending on the plant species, primary dormancy can either be conferred by the embryo or imposed by the surrounding tissues (Finch‐Savage and Leubner‐Metzger, 2006). The latter is known as coat‐imposed dormancy and is prevalent in the Brassicaceae, including Arabidopsis. Coat‐imposed dormancy requires properties of both the seed coat and endosperm in Arabidopsis (Debeaujon *et al*., 2000; Bethke *et al*., 2007; Doherty and Kay, 2010; Lee *et al*., 2010, 2012b; Piskurewicz and Lopez‐Molina, 2016; Fedi *et al*., 2017).

The endosperm is also an important site for ABA signalling in seeds, and ABA transport from the endosperm to the embryo is associated with the prevention of germination in dormant seeds (Lee *et al*., 2010; Kang *et al*., 2015; Chahtane *et al*., 2016). Furthermore, the endosperm may also be the site of perception of environmental signals that regulate seed dormancy and germination. For instance, phytochrome activity in the endosperm is sufficient to regulate germination (Lee *et al*., 2012a) and the temperature‐regulated and dormancy‐inducing *MOTHER OF FT AND TFL1* (*MFT*) gene is only expressed in the endosperm during seed development (Vaistij *et al*., 2013). Furthermore, DELAY OF GERMINATION 1 (DOG1) activity in the endosperm is sufficient for the control of dormancy (Graeber *et al*., 2014). Taken together, an emerging paradigm is that, at least in the case of Arabidopsis, the endosperm plays a key role in primary control of dormancy. Much of the endosperm is consumed before the switch to seed maturation, making space for the embryo to expand and accumulate storage reserves (Fourquin *et al*., 2016). Endosperm consumption is triggered by the pressure exerted by the surrounding seed coat, but also requires the activity of a heterodimeric complex of two closely related basic helix–loop–helix transcription factors ZHOUPI (ZOU) and INDUCER OF CBF EXPRESSION1 (ICE1) (Denay *et al*., 2014; Fourquin *et al*., 2016). Consistent with the available *in silico* data (Le *et al*., 2010), expression analysis shows that *ZOU* is endosperm‐specific (Yang *et al*., 2008) and *ICE1* is expressed in endosperm and to lower levels in embryo and testa, with strong expression in the embryo‐surrounding endosperm (Denay *et al*., 2014). As expected, both *ice1* and *zou* mutants retain an excess of endosperm material at maturity and development of the embryo is restricted, although major embryo tissues differentiate and seeds remain viable (Yang *et al*., 2008; Denay *et al*., 2014). The behaviour of *ice1* seedlings is not completely normal; Liang and Yang (2015) demonstrated that *ice1* mutant seeds exhibit a sugar‐dependent seedling growth phenotype and hypersensitivity to ABA and high glucose.

ICE1 has multiple functions in plants including regulation of cold acclimation and stomatal lineage development (Chinnusamy *et al*., 2003; Agarwal *et al*., 2006; Miura *et al*., 2007; Zhu *et al*., 2011; Kim *et al*., 2015). The target genes of ICE1 in cold signalling, the *C‐REPEAT BINDING FACTORS* (*CBF*s), are also necessary for normal seed dormancy but are not temperature‐regulated in seeds (Kendall *et al*., 2011). In contrast *ZOU*, also known as *RETARDED GROWTH OF EMBRYO1 (RGE1)* is only expressed in the endosperm, where it regulates the expression of genes necessary for endosperm breakdown and embryonic surface formation (Kondou *et al*., 2008; Yang *et al*., 2008; Xing *et al*., 2013; Moussu *et al*., 2017).

Here we show that the *ice1* and *zou* mutants have increased dormancy accompanied by increased ABA levels in the mature seeds. During late embryogenesis and in mature seeds, ICE1, which is present in the endosperm, inhibits expression of the transcription factor *ABA INSENSITIVE 3*, which itself is a central player in the formation of dormant seeds (Giraudat *et al*., 1992) and prevention of germination (Giraudat *et al*., 1992; Nambara *et al*., 1992). Our data therefore show that, in Arabidopsis, ICE1 and ZOU act during maturation to determine the depth of primary dormancy independently of their effect on endosperm development.

## Results

**Figure 1:**
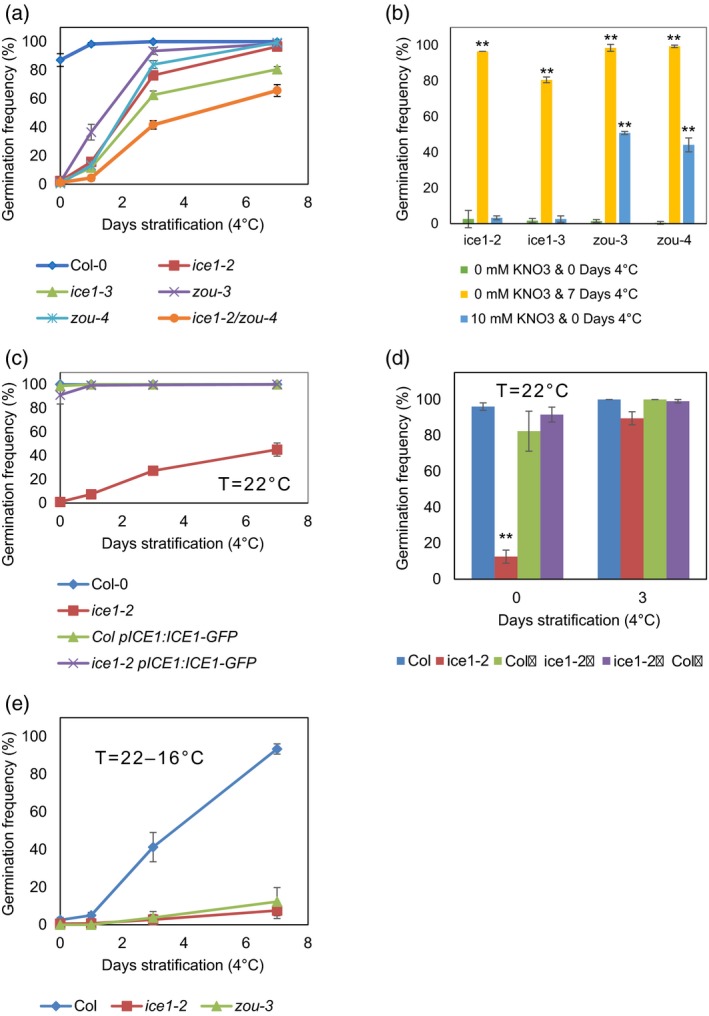
Loss of *ice1* or *zou* from the endosperm results in increased dormancy, where homodimers as well as heterodimers may both play a role. (a) The germination frequency for seeds of the wild type (Col‐0, blue diamonds), *ice1‐2* (red squares), *ice1‐3* (green triangles), *zou‐3* (purple crosses), *zou‐4* (cyan asterisks) and the *ice1‐2*/zou‐4 double mutant (orange circles) matured at 22°C without or with stratification at 4°C for the given times. (b) The germination frequency of freshly harvested seeds of *ice1‐2*,* ice1‐3*,* zou‐3* and *zou‐4* matured at 22°C (green bars) compared with stratification at 4°C for 7 days (yellow bars) or without stratification but with 10 mm potassium nitrate included in the water agar (blue bars). (c) The germination frequency for seeds of the wild type (Col‐0, blue diamonds), *ice1‐2* (red squares), wild type expressing ICE1‐GFP under its own promoter (Col *pICE1:ICE1‐GFP*, green diamonds) or *ice1‐2* expressing ICE1‐GFP under its own promoter (*ice1‐2 pICE1:ICE1‐GFP*, purple crosses). (d) The germination frequency for seeds of the wild type (Col‐0 blue bars), *ice1‐2* (red bars) and reciprocal crosses with wild‐type maternal crossed by *ice1‐2* pollen (green bars) or *ice1‐2* maternal crossed by wild‐type paternal (purple), without or with stratification for 3 days at 4°C. (e) The germination frequency of freshly harvested seeds matured at 16°C of the wild type (Col‐0, blue diamonds), *ice1‐2* (red squares) and *zou‐3* (green triangles) without or with stratification at 4°C for the given times. For (a), (b), (c) and (e), data are averages of five biological replicate seed batches with at least 45 seeds per batch ± SE. For (d), data are averages of five or more biological replicates of Col‐0 or *ice1‐2*, respectively, with at least 20 seeds per batch or six Col♀ ice1‐2♂ or eight ice1‐2♀ Col♂ individual siliques with an average of 15 seeds per silique ± SE. For all, significant differences by Student's *t*‐test on arcsine‐transformed germination data are shown where *P < 0.05; **P < 0.01.

Loss of *ice1* or of *zou* clearly led to reduced germination of newly produced seeds (Figure 1). These phenotypes were robust and the differences between the mutants and wild type were seen across multiple experiments, as demonstrated in Figure S1 in the online Supporting Information; this statement is supported by the statistical analysis in Table S2. To demonstrate that this phenotype indeed increased dormancy levels, we determined what effect dormancy‐breaking treatments would have on the wild type and *ice1* and *zou* mutants. Stratification promoted the germination of two alleles of *ice1* and two alleles of *zou* (Figure 1a,b). As ZOU and ICE1 are known to form hetero‐ and homo‐dimers (Denay *et al*., 2014), we investigated dormancy in the *ice1‐2 zou‐4* double mutant. The latter was more dormant than either *ice1‐2* or *zou‐4* single mutants (Figure 1a). Although 7 days of cold stratification was sufficient to significantly promote germination of all four mutants, the application of exogenous potassium nitrate only promoted the germination of the two alleles of *zou* but not the *ice1* alleles (Figure 1b). Furthermore, the application of exogenous gibberellic acid (GA_3_), which is a hormone that is able to promote germination of most dormant Arabidopsis seeds, was sufficient to promote germination of freshly harvested *ice1* or *zou* seeds, as was after‐ripening (Figure S2). These data suggest that, in addition to the morphological phenotype, *ICE1* and *ZOU* have a role in control of seed dormancy.

The increased dormancy effect of *ice1* was complemented when we crossed *ICE‐GFP* under its own promoter (*pICE1*:*ICE1‐*GFP; Figure 1c; Denay *et al*., 2014) into the *ice1‐2* background (*ice1‐2 pICE1*:*ICE1‐GFP* Figure 1c). As predicted from *in silico* data (Le *et al*., 2010) and previous expression (Denay *et al*., 2014) and localisation studies (Kanaoka *et al*., 2008), we observed the GFP signal in the stomata of leaves and the endosperm of developing seeds in *ice1‐2* expressing *pICE1:ICE1‐GFP* (Figure S3). The *ice1* dormancy phenotype is not inherited maternally, as the heterozygotes demonstrate a wild‐type phenotype regardless of whether the *ice1* is of maternal or paternal origin (Figure 1d). Therefore, we concluded that ICE1 activity in the Arabidopsis endosperm was necessary for normal control of seed dormancy and that both paternal and maternal copies contributed to this process.

Lowering the temperature during seed maturation is sufficient to increase levels of seed dormancy (MacGregor *et al*., 2015). *ICE1* has been implicated in the response to and propagation of the cold signalling response (Chinnusamy *et al*., 2003; Miura *et al*., 2007; Kim *et al*., 2015). We therefore determined whether *ICE1* or *ZOU* were required for the response to low temperatures during seed maturation. Both *ice1‐2* and *zou‐3* responded to this decrease in maturation temperature and, like the wild type, exhibited increased dormancy (Figure 1e). Therefore, increased dormancy in response to decreased temperature is independent of *ICE1* and *ZOU*.

**Figure 2:**
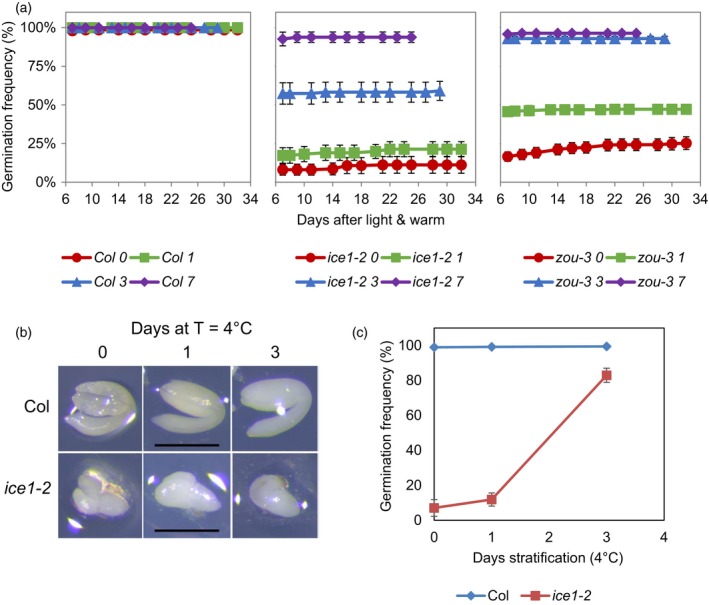
The altered germination frequency of *ice1* and *zou* are not an indirect consequence of retarded embryo morphology that can be rectified by long germination periods or cold stratification. (a) The germination frequency for freshly harvested wild‐type (Col‐0), *ice1‐2* and *zou‐3* seeds matured at 22°C without (red circles) or with stratification for 1 (green squares), 3 (blue triangles) or 7 (purple diamonds) days. Data are averages of five or more biological replicate seed batches with at least 20 seeds per batch ± SE. (b) Morphology of wild‐type (Col‐0) or *ice1‐2* embryos dissected from seeds with 0, 1 or 3 days of stratification. (c) The germination frequency for freshly harvested wild‐type (Col‐0, blue diamonds) or *ice1‐2* (red squares) from seeds out of which the embryos in (b) were dissected. (b). Data are averages of five or more biological replicate seed batches with at least 15 seeds per batch ± SE.

*ice1* and *zou* exhibit abnormal seed development including arrest of the endosperm developmental programme at the fully cellularised stage and the resultant mechanical restriction of embryo development (Denay *et al*., 2014). We therefore considered whether the alterations to dormancy we observed were an indirect consequence of these changes. For instance, retarded embryo development and a larger endosperm to penetrate could cause the germination programme to run slowly or not at all. To determine whether the seeds were truly dormant or simply slow to germinate, we assessed germination of *ice1* or *zou* for 30 days in seeds with or without cold stratification treatments. In these extended germination experiments we observed little or no extra *ice1* or *zou* mutant seed germination after 7 days without stratification (Figure 2a). This shows that the mutant embryos are not defective in the germination process itself but rather germinate to low levels due to an increase in seed dormancy levels. Because of the morphological retardation of embryo development in *ice1* and *zou* we tested whether *ice1* seeds had acquired an additional morphological dormancy that was released by stratification. We found that stratification caused no change to *ice1* mutant embryo morphology or development, but was sufficient to release dormancy, demonstrating that the increased dormancy in *ice1* is physiological (Figure 2b,c).

**Figure 3:**
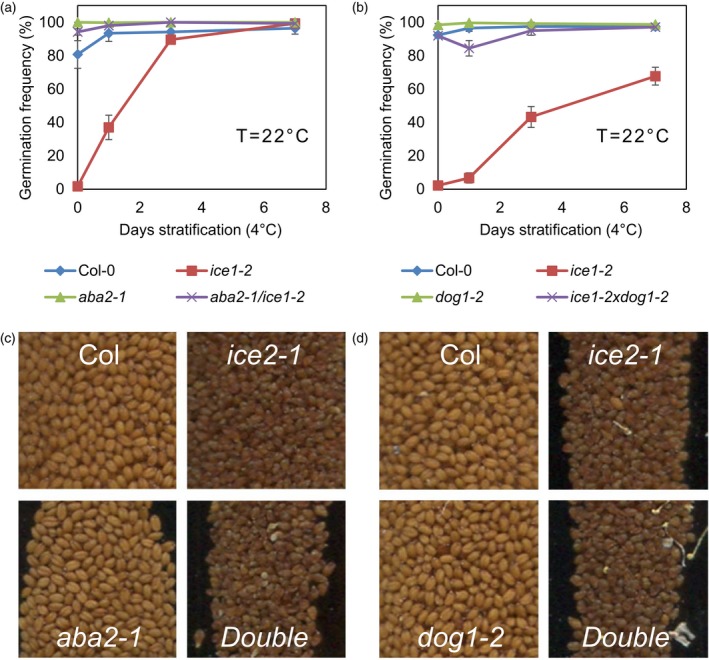
Abscisic acid biosynthesis and *DOG1* are required for the seed dormancy phenotype, but not the seed morphology phenotype, of *ice1*. (a) The germination frequency for seeds of the wild type (Col‐0, blue diamonds), *ice1‐2* (red squares), *aba2‐1* (green triangles) and the *aba2‐1/ice1‐2* double mutant (purple crosses) matured at 22°C without or with stratification at 4°C for the given times. (b) The germination frequency for seeds of the wild type (Col‐0, blue diamonds), *ice1‐2* (red squares), *dog1‐2* (green triangles) and the *dog1‐1/ice1‐2* double mutant (purple crosses) matured at 22°C without or with stratification at 4°C for the given times. For (a) and (b), data are averages of five or more biological replicate seed batches with at least 50 seeds per batch ± SE. (c), (d) Fifty‐millimetre squares showing representative seeds from (a) and (b).

To further test whether seed dormancy in *ice1* and *zou* is physiological we crossed *ice1‐2* to the *abscisic acid deficient 2* (*aba2‐1*) mutant and to *dog1‐2*, noting that DOG1 activity in the endosperm is sufficient to confer seed dormancy (Graeber *et al*., 2014). Both the *ice1‐2 aba2‐1* and *ice1‐2 dog1‐2* double mutants showed high germination frequencies, reversing the stronger dormancy of the *ice1‐2* mutant (Figure 3a,b). Although non‐dormant, the double mutant seeds between *aba2* or *dog1* and *ice1* still exhibited the darker shrivelled seed phenotype and altered embryo morphology characteristic of *ice1* (Figure 3c,d). These data further support the conclusion that the failure of *ice1* mutant seeds to germinate is not directly related to the defect in embryo development, because seeds exhibiting the *ice1*/*zou* morphological phenotype are capable of normal germination rates. Taken together, our data show that *ICE1* is necessary for normal seed dormancy and acts in the endosperm in a manner dependent on both ABA and *DOG1* to affect the germination of primary dormant seeds. This effect is genetically separable from the role in endosperm developmental transitions.

**Figure 4:**
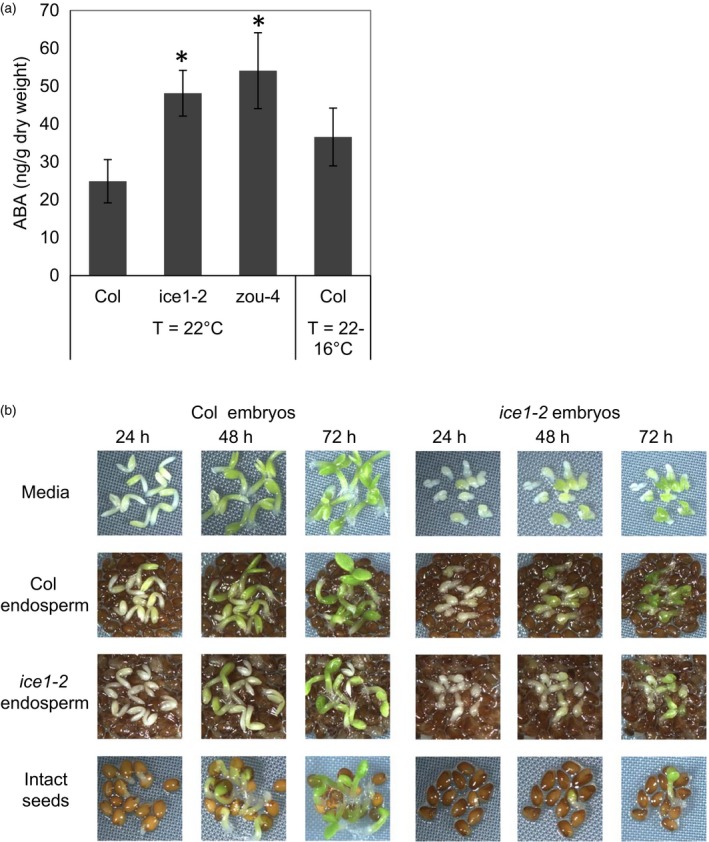
Mature *ice1* and *zou* seeds contain more ABA and the *ice1* endosperm is necessary and sufficient to slow the greening of excised embryos. (a) Measurements of ABA from freshly harvested seed from four or more biological replicates of wild‐type (Col‐0), *ice1‐2* or *zou‐4* seeds matured at 22°C or the wild type (Col‐0) matured at 16°C. Significant differences by Student's *t*‐test are shown where *P < 0.05; **P < 0.01. (b) Seed coat bedding assay using wild‐type (Col‐0) or *ice1‐2* embryos on water agar, Col‐0 endosperm or *ice1‐2* endosperm photographed every 24 h for 72 h. Intact seeds of each genotype sown on water agar are shown for reference.

Production of ABA by the endosperm is known to be a critical step in repressing the germination of dormant seeds upon their imbibition (Lee *et al*., 2010; Kang *et al*., 2015) and *ice1‐2* mutants showed an ABA‐dependent increased seed dormancy phenotype (Figure 3). To determine if there were altered levels of ABA in the *ice1* and *zou* mutants, we measured the ABA content of mature seeds (Figure 4a). Consistent with the increase in seed dormancy, both mutants had a higher ABA content in the mature seed compared with the wild type (Figure 4a). To test whether the increase in seed ABA was being produced by the endosperm, we used a previously described seed coat bedding assay (SCBA) (Lee *et al*., 2010); Figure 4c). Wild‐type and *ice1‐2* embryos were slower to green on a bed of *ice1‐2* endosperms than on an equivalent bed of wild‐type endosperms (Figure 4c). Furthermore, the greening rates of wild‐type and *ice1‐2* embryos were similar, suggesting that embryo ABA content and signalling were not substantially dissimilar between the two genotypes. Taken together, our results suggest that *ICE1* activity affects seed dormancy through endospermic ABA production.

**Figure 5:**
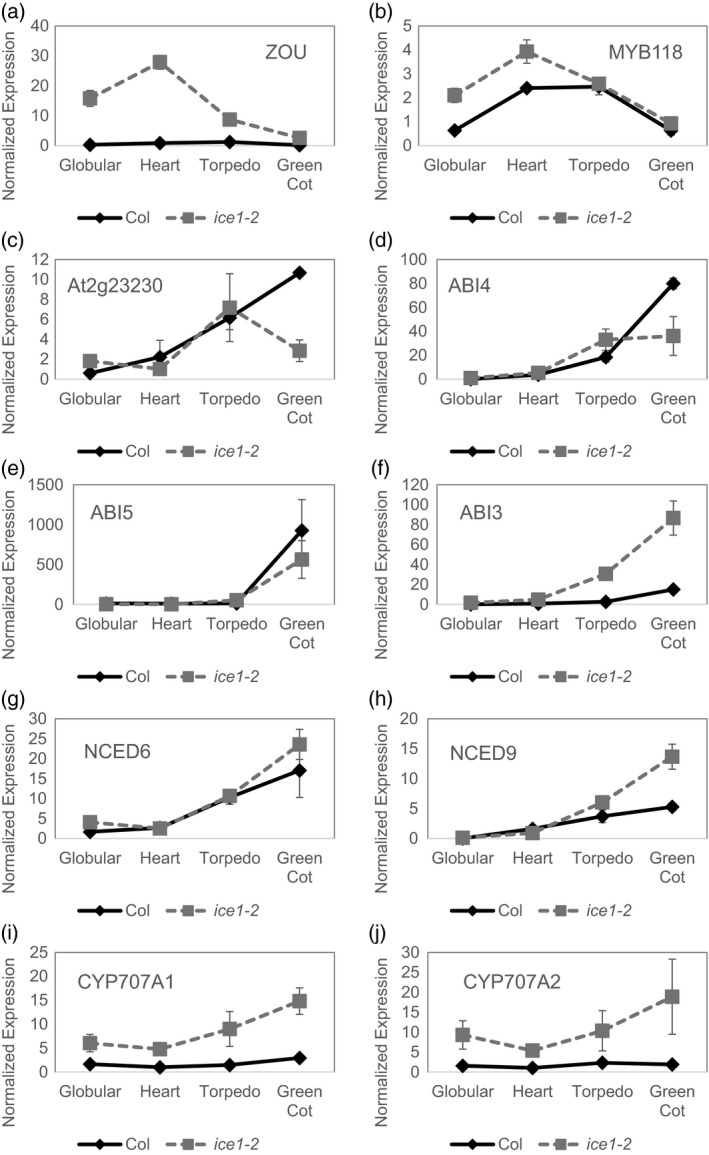
*ice1* changes the expression of many genes including some in ABA biosynthesis, catabolism and/or signalling; however only *ABA INSENSITIVE 3* is significantly misregulated in developing *ice1* seeds. Wild‐type or *ice1* cDNA from developing seeds at globular, heart, torpedo or green cotyledon (Green Cot) stage were examined using quantitative PCR for the expression of *ZHOUPI* (*ZOU*)*, MYB118*, the embryo‐specific *At2g23230*,*ABSCISIC ACID INSENSITIVE4* (*ABI4*), *ABSCISIC ACID INSENSITIVE5* (*ABI5*), *ABSCISIC ACID INSENSITIVE3* (*ABI3*), the 9‐*cis*‐epoxycarotenoid dioxygenases *NCED6* and *NCED9* and the abscisic acid 8′‐hydroxylases *CYP707A1* and *CYP707A2* that were normalised against a clathrin adaptor complex subunit (CACS, At5g46630; Nelson *et al*., 2009). Similar data were found for normalisation against the control gene *At4g12590* (Saez‐Aguayo *et al*., 2017). The wild type (Col) is represented as black diamonds and *ice1‐2* as grey squares with a hatched line. Data are averages of three biological replicate seed batches ± SE.

ICE1 is a basic helix–loop–helix transcription factor and has been shown to bind to promoter elements and alter gene expression (Chinnusamy *et al*., 2003; Agarwal *et al*., 2006; Zhu *et al*., 2011). To investigate the mechanism(s) through which ICE1 regulates ABA responses, we examined the expression levels of relevant genes in developing seeds of *ice1* compared with the wild type. Understanding how transcripts are regulated by ICE1 in whole seeds is complicated by the fact that *ice1* not only has a potentially direct affect on regulation of gene expression but also, because of the aberrant endosperm consumption that occurs after the heart stage (Denay *et al*., 2014), the embryo to endosperm ratio is altered in these mutants. Therefore, it is reasonable to expect a general over‐representation of endosperm‐expressed transcripts in *ice1* mutant seeds. Thus, we first examined the expression of endosperm‐ and embryo‐specific markers in wild‐type and *ice1‐2* mutant seeds (Figure 5). The transcripts of endosperm‐expressed *ZOU* (Kondou *et al*., 2008; Yang *et al*., 2008) and *MYB118* (Barthole *et al*., 2014) were more highly expressed in *ice1‐2* during the early stages of development (Figure 5a,b). The development of wild‐type and *ice1* seeds is visually comparable until the heart stage of development (Denay *et al*., 2014), so these data suggest that ICE1 affects the transcript levels of both genes. Conversely, the embryo‐expressed genes *At2g23230* (Le *et al*., 2010) and *ABSCISIC ACID INSENSITIVE4* (*ABI4*) (Penfield, 2006) were expressed at a similar level in wild‐type and *ice1‐2* mutant seeds until the cotyledon stage, at which point expression was lower in *ice1‐2* (Figure 5c,d). These expression patterns are consistent with the reduced embryo–endosperm ratio in *ice1* in the later developmental stage and suggest that indirect effects of ICE1 on transcription caused by alterations in seed development are only likely to be observed after the torpedo stage of seed development in our analysis.

The genes *ABI3* and *ABI5* encode transcription factors with key roles in ABA signalling in seeds (Koornneef *et al*., 1984; Giraudat *et al*., 1992; Finkelstein and Lynch, 2000; Lopez‐Molina and Chua, 2000). In the wild type both genes are expressed in the embryo and endosperm (Penfield, 2006). Loss of *ICE1* does not have a significant effect on the expression of *ABI5* (Figure 5e), but *ice1‐2* exhibits increased *ABI3* expression compared with the wild type at all stages after the heart stage (Figure 5f). Because ABI3 is an important dormancy‐inducing protein, unlike ABI5, the increase in expression observed in *ice1* may be important for the observed changes in dormancy, especially as they are accompanied by changes in ABA levels (Figure 4).

The two 9‐*cis*‐epoxycarotenoid dioxygenases NCED6 and NCED9 are required for the catalysis of the first step of ABA biosynthesis from carotenoids (Iuchi *et al*., 2001; Lefebvre *et al*., 2006). In wild‐type seeds, *NCED6* is expressed in the endosperm during seed development (Lefebvre *et al*., 2006), although more recent transcriptome analysis shows *NCED6* mRNA to be present mainly in the seed coat (Le *et al*., 2010). Although *NCED9* is present in the peripheral layers of both the endosperm and the embryo, its expression during early stages of development is in the outer integument layer 1 of the testa and is confined to epidermal cells of the embryo after mid‐development (Le *et al*., 2010; Frey *et al*., 2012). The loss of *ICE1* does not affect *NCED6* expression (Figure 5g) while *NCED9* is increased in *ice1* during the later stages of development (Figure 5h). This increase in the ABA‐biosynthetic *NCED9* is consistent with the increased ABA content observed in *ice1* seeds (Figure 4).

We also investigated two ABA 8′‐hydroxylases, *CYP707A1* and *CYP707A2*. Of the major transcripts encoding enzymes with roles in ABA metabolism, *CYP707A1* is the only one predominantly expressed in wild‐type endosperm tissue during mid‐maturation (Okamoto *et al*., 2006). In the wild type, *CYP707A2* is expressed in the embryo and the endosperm during late maturation through to germination and is responsible for the regulation of ABA levels during late maturation to germination (Okamoto *et al*., 2006). Expression of both *CYP707A1* and *CYP707A2* was higher in *ice1‐2* than the wild type at all time points (Figure 5i,j). This is not consistent with this effect being associated with dormancy change in *ice1‐2*, because high *CYP707A* expression is associated with low dormancy in wild‐type seeds (see, for example, Kendall *et al*., 2011). This is instead consistent with the fact that the expression of these genes is induced by ABA (Kushiro *et al*., 2004) and *ice1‐2* seeds have elevated ABA levels (Figure 4). We therefore concluded that this effect must be secondary to the elevated ABA content rather than due to a direct effect of *ice1*.

**Figure 6:**
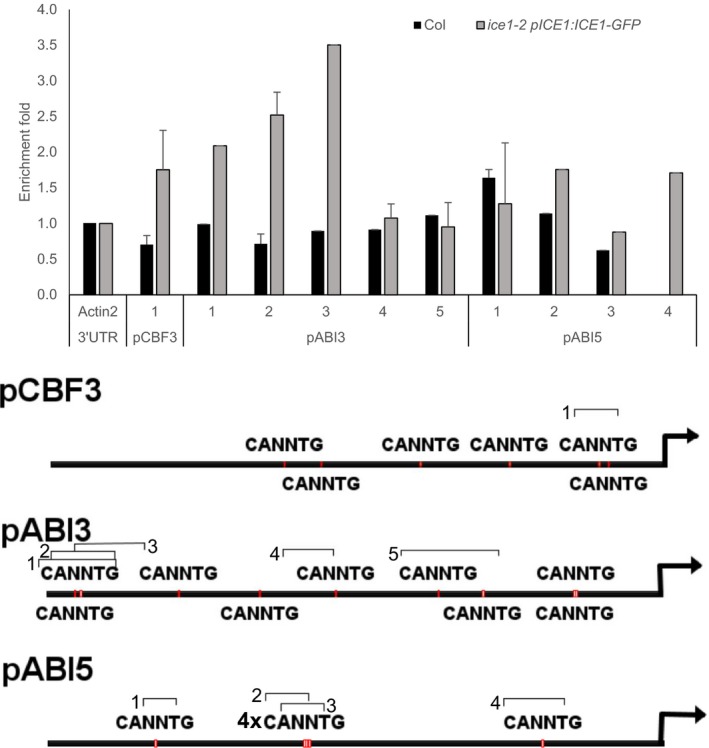
Chromatin immunoprecipitation results. Chromatin immunoprecipitation using endosperm‐enriched fractions of *ice1‐2 pICE1:ICE1‐GFP* (grey bars) shows enrichment at regions of the *ABI3* promoter that contain putative ICE1‐binding sites. This enrichment is not seen in the wild type (Col‐0, black bars). Data represent the average ± SE of three biological replicates per locus. Primers in the 3′ untranslated region (3′UTR) of ACTIN2 (from Adams *et al*., 2015) were used as a negative control and in the promoter of CBF3 as a positive control. The lower panel represents the *ABI3* and *ABI5* promoters with the quantitative PCR targets and putative ICE1‐binding sites indicated.

ICE1 is a transcriptional activator with demonstrated DNA‐binding capabilities and has been shown to bind to MYC recognition sites (5′‐CANNTG‐3′) found in the CBF3/DREB1A and *BON1‐ASSOCIATED PROTEIN1* promoters (Chinnusamy *et al*., 2003; Lee *et al*., 2005; Agarwal *et al*., 2006; Zhu *et al*., 2011). Therefore, we wanted to determine if there was any evidence for direct binding of ICE1 to the ABA genes investigated above. We searched the promoters of these genes for putative ICE1‐binding sites and found several candidate locations in the *ABI3* promoter (Yilmaz *et al*., 2010); File S1). Chromatin immunoprecipitation (ChIP) on endosperm‐enriched fractions of mature *ice1‐2 pICE1:ICE1‐GFP* or wild‐type (Col‐0) seeds were used to test for evidence of the association of ICE1 with the *ABI3* promoter. As a control, we analysed the *ABI5* promoter because *ABI5* expression in seeds was not affected by *ice1‐2* (Figure 5). No evidence was found for GFP enrichment at the promoter of *ABI5* or with the other negative controls (Figure 6). We also found no evidence for enrichment at putative ICE1‐binding sites in the promoters of *CYP707A2*,* CYP707A1*,* NCED6* or *NCED9* (Figure S4). However, the *ice1‐2 pICE1:ICE1‐GFP* line demonstrated enrichment over the wild type at the *CBF3* promoter, as expected from Chinnusamy *et al*. (2003), as well as at three locations in the promoter of *ABI3* (Figure 6). This area is approximately 2 kb upstream of the *ABI3* translation start site and coincides with a cluster of putative *cis*‐elements that strongly resemble those previously identified as ICE1‐binding sites (Chinnusamy *et al*., 2003; Kim *et al*., 2015). ICE1 is enriched at the *ABI3* promoter in a region containing the sequence of previously described *cis*‐elements that are bound by the ICE1 protein *in vitro*. Loss of *ICE1* leads to high levels of *ABI3* transcript, so we therefore conclude that ICE1 represses *ABI3* transcription. Because the *AFL* transcription factors directly upregulate ABA synthesis in Arabidopsis seeds (Gazzarrini *et al*., 2004), our data suggest that ICE1 promotes dormancy through modulation of the levels of *AFL* transcription factor in the endosperm.

## DISCUSSION

**Figure 7:**
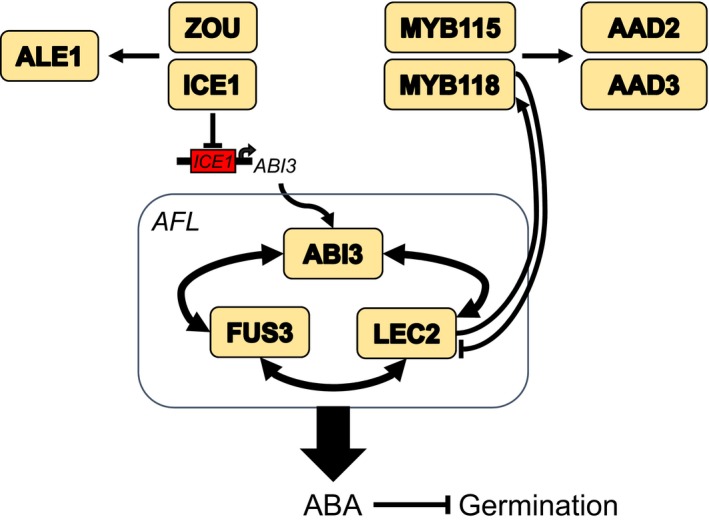
Model summarising how repression of the *AFL* transcription factor *ABI3* by ICE1 and ZOU will regulate ABA metabolism in the endosperm. *In endosperm, ICE1 is enriched at the ABI3* promoter and represses its expression. The *AFL* transcription factors, which are maximally expressed in the developing endosperm (Le *et al*., 2010), upregulate ABA synthesis in Arabidopsis seeds. ABA is necessary and sufficient to repress germination. The *AFL* transcription factors act by regulating each other's expression and are necessary for dormancy establishment. ZOU and ICE1 are also involved in regulating *ALE1* and therefore embryonic cuticle formation (Denay *et al*., 2014). This parallels the activity of MYB115/MYB118, which in addition to regulating fatty acid biosynthesis through the ∆9 acyl‐ACP desaturases AAD2 and AAD3 (Troncoso‐Ponce *et al*., 2016) also inhibit endosperm maturation via the *ALF* transcription factor *LEC2* (Barthole *et al*., 2014). LEC2 is also a transcriptional activator of *MYB118* (Barthole *et al*., 2014).

The acquisition of seed dormancy has allowed plants to establish seed banks and correctly time their germination according to seasonal cues. We demonstrate herein that loss of function of *ICE1* and/or *ZOU* results in seeds with increased primary dormancy and elevated accumulation of ABA (Figures 1, 2 and 4). The characterisation of the dormancy effects of ICE1 and ZOU is complicated by the co‐occurrence of the effects on seed development caused by the failure of endosperm consumption. However, we show that the two processes can be separated. The aberrant endosperm consumption alone is insufficient to explain the dormancy phenotype, because in the *aba2* and *dog1* mutant backgrounds normal germination is restored without an effect on seed morphology (Figure 3). Our data show that the increase in dormancy is associated with an increase in seed ABA levels and that this ABA is probably present in the endosperm (Figure 4). The SCBA data demonstrate that mature *ice1* endosperm works more efficiently to arrest embryonic growth (Figure 4), which is consistent with the idea that this is a mature endosperm that has higher ABA levels. This view is further supported by the fact that both *ICE1* and *ZOU* are expressed in the endosperm of seeds and bolsters the increasing body of evidence demonstrating that the endosperm is the primary site of control of dormancy and germination in Arabidopsis. Our data show that the *AFL* transcription factor gene *ABI3* is a direct target of ICE1 in seeds (Figure 6) and *ABI3* transcript levels are higher in *ice1* seeds than in the wild type (Figure 5). A similar effect of *ICE1* on *ABI3* levels has been observed in seedlings on high‐sugar media (Liang and Yang, 2015). Transcript levels of some endosperm‐expressed *AFL* target genes such as *MYB118* (Barthole *et al*., 2014) are also increased in *ice1* (Figure 5). Our data are therefore consistent with a model in which *ICE1* and *ZOU* are inhibitors of the seed maturation programme in the endosperm via control of *AFL* activity, as well as promoters of endosperm consumption and biogenesis of embryonic cuticle via *ABNORMAL LEAF‐SHAPE 1* (*ALE1*; Denay *et al*., 2014). This role is very similar to that described previously for *MYB118*. This transcription factor, which is closely related to MYB115 (Wang *et al*., 2009), functions in the endosperm and is essential for biosynthesis of omega‐7 monounsaturated fatty acids via transcription of two ∆9 acyl‐ACP desaturases, *AAD2* and *AAD3* (Troncoso‐Ponce *et al*., 2016), and inhibits *AFL* gene activity, thus delaying the seed maturation programme (Barthole *et al*., 2014; Figure 7).

Mature angiosperm seeds display considerable morphological diversity, and this is accompanied by a range of dormancy‐inducing mechanisms. For instance, in morphological dormancy seed dormancy is initiated by an arrest of embryo development before maturation, such that further development is necessary after shedding before the seed can germinate. There are also examples of seeds displaying two distinct types of dormancy, especially combining morphological dormancy with physiological dormancy, each of which may be responsive to distinct environmental signals (Baskin and Baskin, 2004). These variations in dormancy programmes appear to be able to evolve independently multiple times, but it is unclear whether or how seed development and physiological dormancy evolve separately or by a common process.

According to the classification of Baskin and Baskin (2014), seeds whose embryos are differentiated but underdeveloped and which exhibit physiological dormancy are classed as having morphophysiological dormancy. The *ice1* and *zou* mutant embryos clearly meet the morphological definition (Denay *et al*., 2014) and the phenotypes of these mutant embryos strongly resemble those from many gymnosperm seeds. During germination, embryo growth takes place before emergence of the shoot and before and during emergence of the root. However, lack of germination is not simply due to delayed embryo growth because prolonged incubation of *ice1‐2* or *zou‐4* seeds does not result in increased levels of germination (Figure 2). Therefore, although *ice1* and *zou* seeds have increased dormancy and altered morphology, they do not exhibit morphological dormancy. To qualify as seeds with morphophysiological dormancy, embryo growth must be a pre‐requisite for either root or shoot emergence and this growth can be promoted by a separate signal from that which breaks the physiological dormancy. We showed that cold does not promote the growth of *ice1‐2* embryos during stratification (Figure 2). In this case, cold is required to break the increased physiological dormancy of *ice1* and embryo growth resumes only after seeds are placed in the warm lit conditions. This behaviour resembles a morphophysiological dormancy state described as ‘non‐deep simple’ (Baskin and Baskin, 2014). *Thalictrum mirabile* (Ranunculaceae) exhibits non‐deep simple dormancy and the seeds require cold stratification followed by warm temperatures which allow embryo growth to resume as the seeds germinate (Walck *et al*., 2011). Regardless of whether *ice1* and/or *zou* seeds exhibit complete morphophysiological dormancy, this raises the prospect that single mutations in key genes can couple physiological dormancy with morphological changes to the embryo in the mature seed, suggesting mechanisms through which the evolution of seed dormancy can occur.

## Experimental Procedures

### Plant material and growth conditions

*Arabidopsis thaliana* (L.) Heynh ecotype Columbia (Col‐0) was used in this study. *ice1‐2* (SALK‐003155; Kanaoka *et al*., 2008) was a kind gift from Keiko Torii. *zou‐4*,* ice1‐2 zou‐4* double mutant and the *pICE1:ICE1‐GFP* in Columbia (Denay *et al*., 2014) were kind gifts from Gwyneth Ingram. *dog1‐2* (Nakabayashi *et al*., 2012) was a kind gift from Wim Soppe. *aba2‐1* (MacGregor *et al*., 2008) was a kind gift from Jocelyn Malamy. Segregating populations of *ice1‐3* (SALK_003426, N503426, not previously characterised) and *zou‐3* (WiscDsLox465F5, N857109, (Zhang *et al*., 2016) were obtained from the Nottingham Arabidopsis Stock Centre and homozygous plants were isolated using standard PCR methods and the primers presented in Table S1. *zou‐3* is in the Col background, despite what is stated elsewhere (Yang *et al*., 2008).

Plants were sown, grown and harvested as per the methods described by MacGregor *et al*. (2015). Great care was taken to ensure that for each figure the controls and mutants were grown together under conditions that were as uniform as possible (e.g. at the same time, in the same tray, on the same shelf, within the same cabinet) so that comparisons between the lines could be made. Dry sterile seeds were sown out and stratified at 4°C for 2–4 days on MS agar plates [4.4 g L^−1^ MS basal salt mixture (Melford Laboratories, cat. no. M0221, http://www.melford.co.uk/) with 0.9% agar (Sigma Aldrich, cat. no. A1296, http://www.sigmaaldrich.com/)]. Seedlings were grown in growth cabinets at 22°C for 10–14 days with 12‐h:12‐h light:dark cycles before being transplanted to 40‐cell trays containing John Innes Seed Compost. Plants were grown under well‐watered conditions at 22°C under standard long days using fluorescent white light at 80–100 μmol m^−2^ sec^−1^ until bolting or anthesis of the first flowers. Once flowering, plants were transferred to growth cabinets running the same conditions, but with the indicated seed maturation temperatures, and left to set seed until dehiscence began.

### Dormancy assays

Mature dry seeds set under the conditions above were harvested and poorly filled seeds excluded using a 250‐μm sieve (Fisher Scientific, cat. no. 11542153, http://www.thermofisher.com/). These sieved seeds were sown directly onto water–agar (0.9%; Sigma Aldrich, cat. no. A1296) and cold‐stratified at 4°C in the dark using a Panasonic MIR‐154 incubator (Panasonic, https://www.phchd.com/global/biomedical/) for the desired length and/or put directly into a 12‐h:12‐h white light (80–100 μmol m^−2^ sec^−1^):dark light regime at 22°C in a Panasonic MLR growth cabinet (Panasonic) for germination. Exogenous gibberellic acid (Gibberellin A_3_; Sigma Aldrich G7645), 10 mm potassium nitrate, norflurazon (PESTANAL®, Sigma Aldrich 34364) or the appropriate solvent controls were supplements to the molten water agar in the concentrations indicated in the figures. Germination was scored as the emergence of the radicle using a Leica MZ6 stereomicroscope (https://www.leica-microsystems.com/) after 7 days of exposure to warm light incubation, unless otherwise indicated. For each data point, germination frequency (%) was calculated as the percentage of seeds germinating from a minimum of 20 seeds from five biological replicates (which were defined as seeds from different mother plants). Data are shown as averages of the biological replicates ± standard error. If statistics are shown, Student's *t*‐tests were performed on arcsine‐transformed data; a single asterisk indicates significance of *P* < 0.05 and a double asterisk is *P* < 0.01. The germination phenotypes of *ice1* and *zou* are robust. Each experiment was repeated multiple times with comparable results being obtained from different repeats; for clarity only data from one experimental replication are shown.

### Double mutant creation and confirmation

Double mutants between *ice1‐2* and *dog1‐2*,* aba2‐1* or *pICE1:ICE1‐GFP* were obtained by using pollen from homozygous donors to fertilise emasculated homozygous *ice1* plants allowing the F_1_ generation to self and screening the F_2_ seeds for the *ice1* shrivelled seed phenotype. Putative *ice1* homozygotes were then sown on plates supplemented with 1% sucrose and transferred to soil once established for further growth. Homozygosity of both mutations was confirmed by using PCR (primer details in Table S1) in the case of *ice1‐2*,* dog1‐2* and *aba2‐1* or by the ubiquitous presence of GFP fluorescence in the stomata of two generations of seedlings for *pICE1:ICE1‐GFP*. GFP fluorescence of 500–530 nm was visualised using a standardised GFP protocol on a stereo‐dissecting microscope.

### Confocal microscopy

Developing seeds of *pICE1:ICE1‐GFP* in *ice1‐2* were excised from the siliques mounted in water between a microscope slide and coverslip and were visualised on a Leica SP8X confocal microscope using an argon ion laser at 488 nm to excite both GFP and autofluorescence; emission of GFP was collected at 500–530 nm and the autofluorescence at 600–630 nm. A 63×/1.2 water immersion objective lens was used. The Z series in Supplemental Figure 3 were collected at 0.5‐μm intervals. Images were processed using Image J (https://imagej.nih.gov/ij/) in which max projections were made and scale bars added. The composite image was made using Leica LAS X software. The stage of development was verified by chloral hydrate clearing of seeds after microscopy.

### Seed coat bedding assays

Seed coat bedding assays were performed using freshly harvested seeds that had been stored at −80°C until analysis according to the protocols in Lee and Lopez‐Molina (2013).

### Phytohormone assays

Abscisic acid was quantified from five biological replicate batches of 100 mg of freshly harvested dry seeds that were flash frozen in liquid nitrogen and stored at −80°C until analysis. Quantification of hormones was performed by ultraperformance liquid chromatography–mass spectrometry analysis of acidified isopropanol (1% acetic acid) extracts as described previously (Dave and Graham, 2012).

### Analysis of gene expression

Three biological replicates of developing seeds at the stages indicated were dissected out of siliques of wild‐type or *ice1‐2* plants grown at 22°C under conditions above directly into RNAlater (Sigma Aldrich, cat. no. R0901), which was subsequently removed before the seeds were flash frozen in liquid nitrogen and stored at −80°C until required for analysis. The RNA was extracted from these seeds as described previously (Penfield *et al*., 2005) and purified via the clean‐up protocol of the RNeasy Plant RNA isolation kit (Qiagen, cat. no. 74904, http://www.qiagen.com/) according to the manufacturer's protocol. First‐strand cDNA was synthesised with 1 μg of total RNA in 20‐μl reactions using Superscript III Reverse Transcriptase (Invitrogen, cat. no. 18080‐044, http://www.invitrogen.com/) and Oligo(dT)12‐18 (Sigma Aldrich, cat. no. 18418‐012) according to the manufacturer's instructions. Then 180 μl of water was added before the quantitative PCR step. Gene expression analysis was determined in a Bio‐Rad CFX CFX96 instrument (http://www.bio-rad.com/) using the primers indicated in Table S1 and Brilliant III Ultra‐Fast SYBR® Green QPCR Master Mix (Agilent Technologies, cat. no. 600883, http://www.agilent.com/) according to both manufacturer's protocols.

### Chromatin immunoprecipitation

Freshly harvested seeds from wild‐type and *ice1‐2 pICE1:ICE1‐GFP* or wild‐type Col‐0 plants were grown under standard long‐day greenhouse conditions, surface‐sterilised for 3 min in bleach and washed at least four times with sterile water. Sterile seeds were plated onto filter paper in Petri dishes containing 20 μm paclobutrazol (Sigma Aldrich, cat. no. 46046). The Petri dishes were sealed with micropore tape and incubated in a 12‐h:12‐h white light (80–100 μmol m^−2^ sec^−1^):dark light regime at 22°C in a Sanyo MLR growth cabinet (Panasonic) for 24 h. Glass microscope slides were used to squeeze seeds until the embryos were forced from the endosperm and seed coat, all of which were collected in a 50‐ml tube. A fraction enriched in endosperm and seed coat was obtained by spinning these mechanically disrupted seeds at 3000 g for 10 min in 40% sucrose (w/v), which separates embryos from endosperm and/or seed coat and intact seeds. Embryos were discarded and the endosperm‐enriched fractions were rinsed with sterile distilled water to remove the sucrose and fixed in 1% formaldehyde for 10 min under a vacuum. Fixed tissues were quenched with a final concentration of 125 mm glycine under a vacuum for 5 min and rinsed at least three times with sterile distilled water before being flash frozen in liquid nitrogen. Isolation and shearing of chromatin and immunoprecipitation of GFP‐enriched fractions were all performed as described elsewhere (Keily *et al*., 2013) using primers described in Table S1.

## Accession numbers and primer sequences

Sequence data from this article can be found in the Arabidopsis Genome Initiative or GenBank/EMBL databases using the accession numbers *ABA2* (AT1G52340), *ABI3* (AT3G24650), *ABI4* (AT2G40220), *ABI5* (AT2G36270), AT2G23230, *CACS* (At5g46630), *CYP707A1* (AT4G19230), *CYP707A2* (AT2G29090), *DOG1* (AT5G45830), *ICE1* (AT3G26744), *MYB118* (AT3G27785), *NCED6* (AT3G24220), *NCED9* (AT1G78390) and *ZOU* (AT1G49770). The primer sequences used are detailed in Table S1. Primers that have not been previously published elsewhere were designed by hand or using dCaps Finder (http://helix.wustl.edu/dcaps/dcaps.html), QuantPrime (Arvidsson *et al*., 2008) or Primer3 (Koressaar and Remm, 2007; Untergasser *et al*., 2012).

## Author contributions

DRM designed and performed the research, analysed the data and wrote the paper. NZ performed the research and analysed the data. MI designed and performed the research and analysed the data. MC performed the research and analysed the data. AD designed and performed the research and analysed the data. LLM designed the research and wrote the paper. SDP designed the research and wrote the paper.

## Conflicts of interest

The authors have no conflicts of interest to declare.

## Supporting information


**Figure S1.** The dormancy phenotypes of *ice1* and *zou* are repeatable and robust.

**Figure S2.** The increased dormancy of *ice1* or *zou* can be rescued by exogenous gibberellin in a concentration dependent manner or by after‐ripening.

**Figure S3.** ICE1‐GFP is located in the nuclei of both stomata in true leaves and endosperm of developing seeds.

**Figure S4.** Chromatin immunoprecipitation using endosperm‐enriched fractions of *ice1‐2 pICE1:ICE1‐GFP* shows no evidence for enrichment at putative ICE1‐binding sites in the promoters of CYP707A2, CYP707A1, NCED6 or NCED9.

**File S1.** Putative ICE1‐binding sites in the targets in Figure 5.

**Table S1.** Primers used herein.

**Table S2.** Testing the significance of the *ice1‐2* and *zou‐4* dormancy phenotypes over multiple experiments.
