# Supplementary figures and images for: ICE1 and ZOU determine the depth of primary seed dormancy in Arabidopsis independently of their role in endosperm development
Source: Plant J. 2019 Feb 18;98(2):277–90. doi: 10.1111/tpj.14211 (PMC6900779; doi:10.1111/tpj.14211)

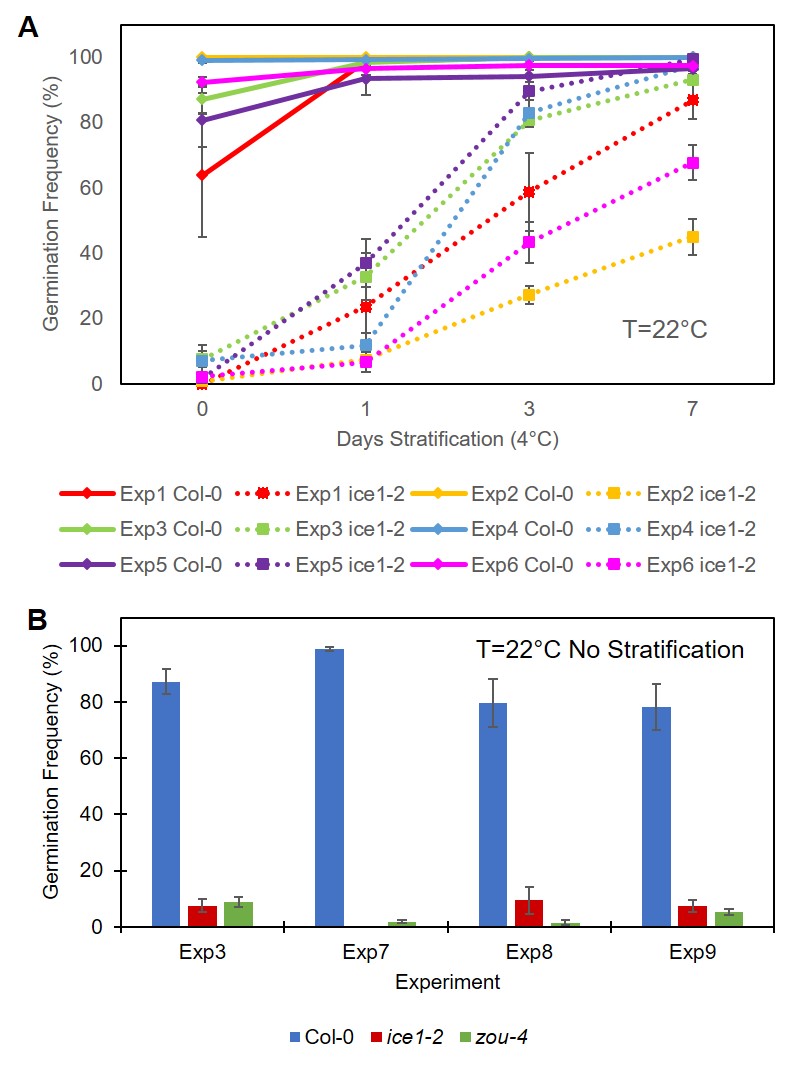

Supplement: Supplementary file 1 — Figure S1. The dormancy phenotypes of ice1 and zou are repeatable and robust. [file TPJ-98-277-s001.jpg]

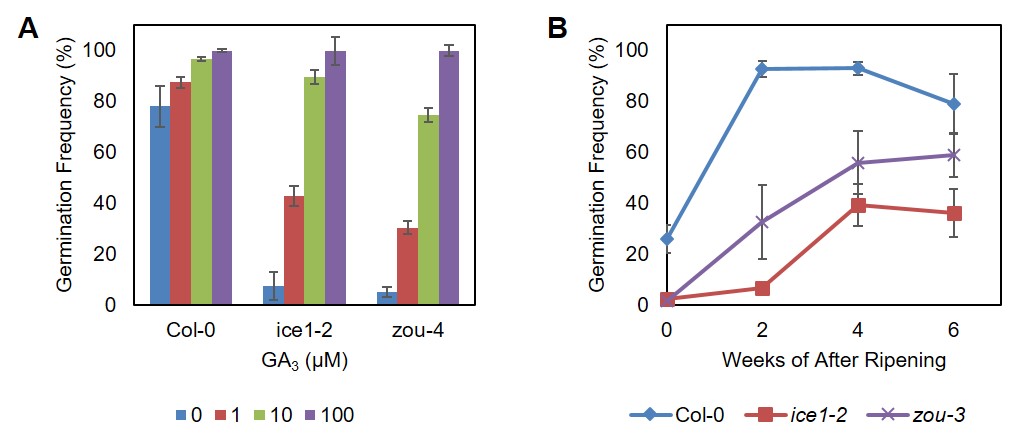

Supplement: Supplementary file 2 — Figure S2. The increased dormancy of ice1 or zou can be rescued by exogenous gibberellin in a concentration dependent manner or by after‐ripening. [file TPJ-98-277-s002.jpg]

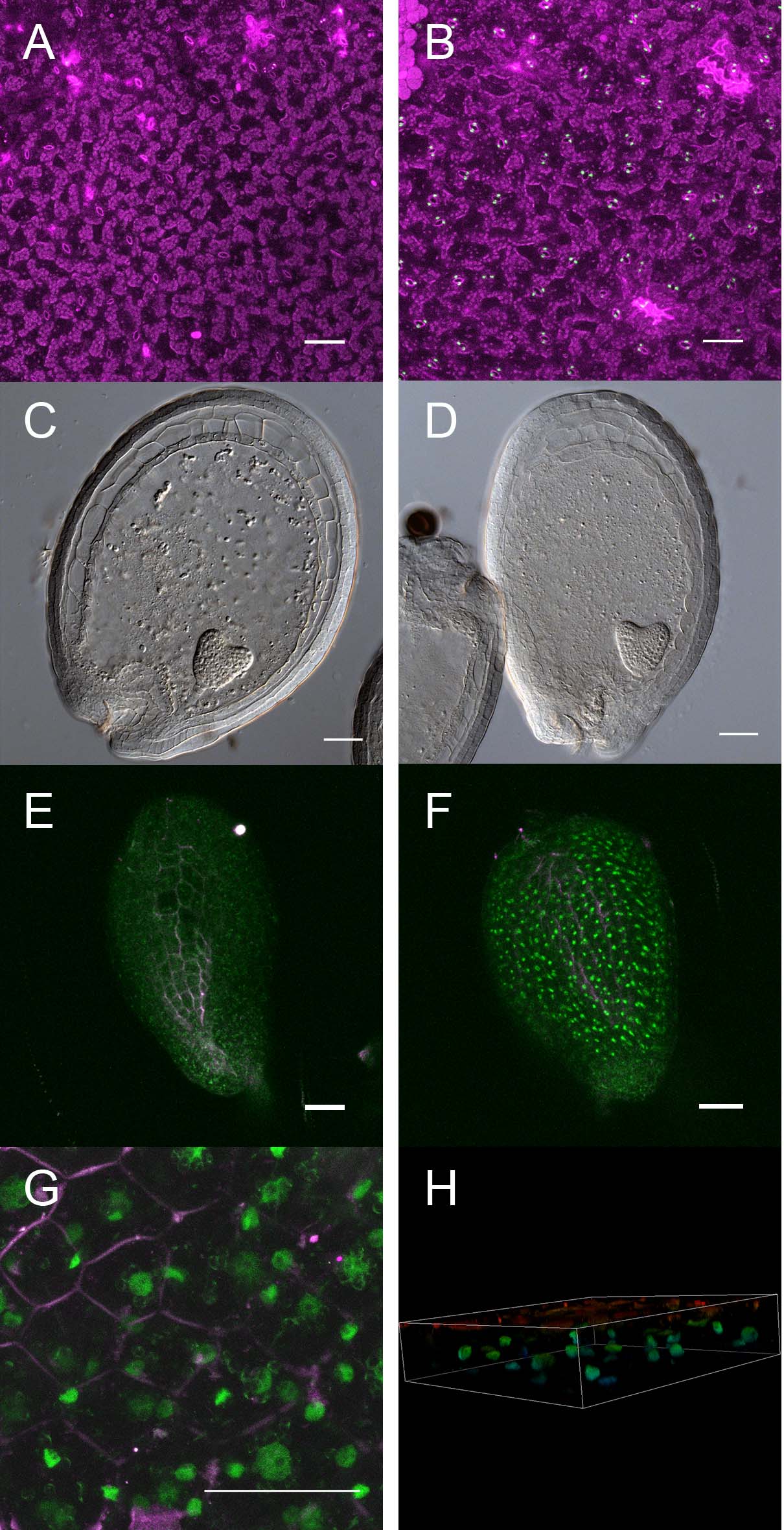

Supplement: Supplementary file 3 — Figure S3. ICE1‐GFP is located in the nuclei of both stomata in true leaves and endosperm of developing seeds. [file TPJ-98-277-s003.jpg]

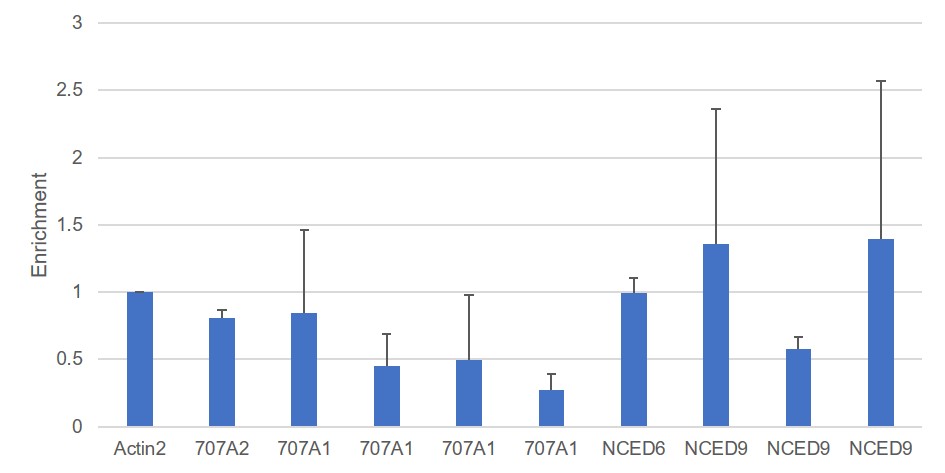

Supplement: Supplementary file 4 — Figure S4. Chromatin immunoprecipitation using endosperm‐enriched fractions of ice1‐2 pICE1:ICE1‐GFP shows no evidence for enrichment at putative ICE1‐binding sites in the promoters of CYP707A2, CYP707A1, NCED6 or NCED9. [file TPJ-98-277-s004.jpg]
