# Supplementary material for: ICE1 and ZOU determine the depth of primary seed dormancy in Arabidopsis independently of their role in endosperm development
Source: Plant J. 2019 Feb 18;98(2):277–90. doi: 10.1111/tpj.14211 (PMC6900779; doi:10.1111/tpj.14211)
Supplement: Supplementary file 5 — File S1. Putative ICE1‐binding sites in the targets in Figure 5. [file TPJ-98-277-s005.pdf]

Supplemental File 1: putative ICE1 binding sites in the targets in figure 5.

Word document showing the locations of the putative ICE1 binding sites in the promoters of the genes used in Figures 5 and 6.

CBF3 promoter

TACAAAACAAATCGTTTTCTACTTCTTTGCTTCACATAAGTTAAAAGTCAAATATT
TAACAAAAAAGATATTAAAAGTCATATTGTAGTTGCTTTCAAGGCAAAATATGTG
GACAGATCATTACACGTGGATGATGTTTGTAAATATGCCACAAAACCTGCATTAC
ATTATTTTATTCTATCTCATGTAAGTTACAGATCTTACAATTTAGCAACAGAAAGC
CACAAAATATTACATAAATTGGCTCGTCTCGAATCTAGCAACCAAAAAAATTCAG
CCCAGTTCACTATAAAGAATATAAAAAAAAGTTTCCTAAAATAGTGTATAAAACC
GAAACAAACTAATTCAACAAACCCGAAATAAACAAATCCGTACGACAACCAAAAA
TATCTTTCAGATGGGTTCCACAAGATAACCCAGTGCCAATCAGAATTCTGAAAG
CGTGGCTCGACCGCGGAAACCATTGTCCATACCTTCTCTTCTTTGTCCCCCCTT
ACGTGGCTCGCTGTGGAGTCTCGTACCACGTGTCGCGTCACTTCACTCTTTACT
TTCTATTTTCCACTAAAATCATAATTTGTCTTTTTCTTGACCATACCCACTCTTTTT
TCTTCTCGTTGTCGTCTTGCTTCTCCTAAATATCTCAAATAACGTGAGAGACTTG
AGTGTGAGAGGTAGGTAACGAGGCAGACTTTTTTTGGAAGCGAATATAACTTAT
GCTGATATTTTATTTAGCTTTCTGATTGGAGTTGAGATATTATATAGGTATTATTG
AGATATTATATACGTATTATTGAGATTTGAGATATTTTGTAGATTTTATAGTTCTAT
CGGACTAATTCTTGGCTTAATCCACTAACATGTTTTTGTTTAGTTAATTAAACTGA
TTATTTTCTGCGCTATAGTTTTGTTAAACACCTTTTAGACGTAACAAAGCAATTAC
GCTTGATCATCCATCGTAGACTCTTTTTCTTTTTTTACATCTCATAGAAGTTTTGT
TTAAACACAGCAGGAAGTAAATTATTTTCTTATTACGTACGTATGATTGTTTTTAG
ACTATTTTAGTACTTTGAGAAGTAAAATTGGGGATACGAGATAAAAGACAATTGA
TTAACATGCTTTTTATTTTGACTTCCGAAACTAATCATGGTTGTCTATGTTTATAA
ATTGTGTTCTTTTTGTTGAAAAACTCAGATAATGATTAAATCAGTAGCGAATGGA
TGGAGAACACATGATTTTAGATTGCATACCGTAAAACAAAAAAAATCATGATGGA
TGTAGAACATTCAAATGGGTCAAATAAATACGTATGTGATCAAAAGAAAGTATGT
GATCAAAAGGGTTAGCACGAGTACCTTGGGAGGAAATTCTTCTAATTATGAATT
ATGCAAGAATTTTCGTCAAGGGAAGGTGGGGAAGAGGTAGCTAAAGAATAGAG
AATCATATGACTAAGGACGTGGTGGTTGAAGGAAATGAGAGAATACATGAAGAA
GAGAAACTTCTTTGAGTGAGAAGGAAGTGCGCTGGCTGGAGAGAAAAGAGAGA
AAAGAGTTTCGAGTGAGAGAGAGGGCGTTGAGATTGTGATCAACTTAATGTAAT
ATGTTCTTTTATTACATTTTCTTTTTGTCATATACTCAAACCTTTTACTATTTTGTC
TCATAAATCTAACACACCCCACCATTTGTTAATGCATGATGGTAGAAAATATTAA
ATATAATTAACTACTTTTATGTGATCAAAATTAGGTTTCAGACTCGTTTCGCGATC
CGATCTACAATTACAACTGCATGCTTCTAATTGATCTAAATTCTAAATTTTTTATA
CATATTAAAAAAACAACTTTTTGTTAAATTCTCAATCATCATTTTTGTGATTAACAA
TTTTTTATAACTCTAAACCAATAATATTTGATTATTTATTTTATATGTATAATGATG
ATTGAGAATTTTAATTAGCAGTCTATTTAGGGTTTTCCTAAAGTTACAATATGTTG
TTACCCTTCTAGTTAAATTTTCCAAAATACCATATTTCATAACTTTTCAAACTGTTT
ATTAATTCAACCGTAAAAAGCACTAAAATGTTACATTTGATCATTCACCCAAATTA

AATTCAAAAGTTTTTCCGCCAAAACTACTTGGTGACTTACGTGCTTATATACGGA
CGACTATTATTATGTTCTATACTTTTTTATACTTTGTTGCACAAATATCTACTCTCC
CAATTCATATTCTAGAAGGATGTGCTATAAGAATGGGAGAAATTACACAAGAAG
AGCATCTTTAAATATCCTCTCACAATCTTTATGTCTAATACACGGGTGAACAATT
AACGACAATTTCTTTATTCAGGAATATAATAATGAATAACGGTTACCCTACACCT
AGTACACTAAATCCTTAACAGCCACACATTCATACGCAAAGAGTTTATAAAACTC
ATAAAGGTATAATAATAACGAGTGAATAAGTCAAAAAAAGTCTTCTCTGGACACA
TGGCAGATCTTAATGAGTGAATCCTTAAACTACTCATTTTACAATTGCTTCGCTG
TGTATAGTTTACGTGGCATTACCAGAGACACAAACTCCGTCTTCGCCTTTTCTTT
TGCCTCTAAAATATCTTCCGCCATTATAAAACAGCATGCTCTCACTCCAACTTTT
ATTTATCTACAAACATTAAATCCACCTGAACTAGAACAGAAAGAGAGAGAAACTA
TTATTTCAGCAAACCATACCAACAAAAAAGACAGAGATCTTTTAGTTACCTTATC
CAGTTTCTTGAAACAGAGTACTCTTCTGATCA

Pair1

CTAACACACCCCACCATTTGTTA

CGAGTCTGAAACCTAATTTTGATCAC

Pair2

GCCACACATTCATACGCAAAGAG

GATCTGCCATGTGTCCAGAGAAG

Pair3

TGACTTCCGAAACTAATCATGGTTG

CATGTGTTCTCCATCCATTCGCTA

ABI3 promoter

AACAAACGACTAGTACTGATATATACATCAATGCTTACAAAAATCAAATTATCTAT
CTCAACGTTTACTTTTGTACGTAATTACAAGTCAATATTAACAAATATTAGTCAAC
TGTAACGGTTGTTGACTCAATTGATACGTGTACGTTTAGGTGGCATGTTTTTTAA
TAAAATAATTTTGTTTTATGTTTTGGGATTAAAAATATTTACTTTTAAAAAACTAAA
CCGAAAAACTGGAACCCCCAACCTCACATATTCATCACCATCGTATCCACAAAC
ATTATCGAGACATAAATAATAATTTATAGATATATAGCAACAAATTGGAATTAAAT
TAATCAAACAATGTCATTTAGAATTGGGGAAATCCTAACCGACGTTATATGATTA
ATTTTAATTCCAATTTATGTATGTGTATATATCTATAAATTATTATTTATATATTGAT
GATGTTCGTGCATGATGGTAATGGTAACCTTTAAAACTTTTGAATCTCAAAACGT
AAAACAACGTCATTTGGTTTGAAGAACATGCCATCTCGACGTACAAGTGTTAGC
TGATTCAACAACCGTTACTGTTAATTACCATAACTAACGTTGACTCATAATTATGT
ACAAAAGTAAGAGTTGAGGTAGATAATTTGAATTTTTTAAATGTTAAAGTATGAA
CATTACTAATTGTTTGTTCAGATTTATAAAACACAGTACCTATTTTGATGTGTTAA
AATGTAATCGGGAAAATAGGAATTTTCCCAAGAAAAAAAAATACACTAAAATTTT
GATGTGTTAAAATGTAATAAATACAAGTACCTATTTCTTTCTACAACAGGTATCAC
ATGATCTGCTCAATCTCATTTTGAAGGCAAGGTCGAACCCTGTTTGTAACTAAAT
ATTTTACAAATACACTAAAAGTTTTGTTTTGTTTTCCTCATGCAGAAAAAAGAAAA
GAAAGAAGAAGAAAAAAAACTCACAAAAACATACATTGTTCTACAATATGTTAAA
GTAATTGTTCTAGGGTAATAAAATCCGTGTCTGCCTCTTCCACTTCAATAGGAAA
GTGTAAGTTTTTCGAATCTTTTTGGCCAGATAATTTAAAACCGACCGAGGAAACT
TACTTTTGTAAAAGTGACCATTTGCACATAGGAAGTAGAAAGTTGATAATTGGAA
AATTTATAATATTTTATACAAAAGAAGTGTTTAAGAACCACCGCTTGGTCTCACC
ACTCAAATTCGAACGTATGGCTCGTGGGCACAAACTCCTGTACCAACCTTTAGA
TACTAGCGGCACGAGGAGACTTCTATTTTCTCTTTGTTTTTAAGTTTTTTTATCGA
CTTAAATATGGCTTTTTGCACCATTTAATTTCAAAACCAATGTCTTTCTCTCGAGG
AACTTTGTTTTTTATTTCTTAGAAGATGAGGGGAGATTTACTATCTAAATAAAATTT
TAAATGTTTGTAAGTATTATGAGCTCAACAATTTTGTCAATAGTGCCACAAATTTA
AACGTTTGCTTTTTGTCTTCTTTGAAAAATCAAATGCTGAAAAACTGTTACATCTC
TTTTTCTTAAAAACTCTTGTCTCTCTCCTTTTCCTTCTGCTGAGGTAATTGAATGC
TGCAAAGAGAAAGAGAATAACTTAAACCCAAAATTACACTTACCGCCAGAAAAA
AAAAAGAGTTCAGTTTAATCTAACATATTTTATACAATACAATTGAATTATATTAG
TAAAAAAAAAACTTCCATATAAATCATGGAACAAACTGGAACACATGGGCTCTCT
TATTTTAATTTATTTTCTTTTTTGAGGGATTTAACCATGTTTATTATATAGTTTTATA
AATATATATATACCATCTCTCCATAATTTATAAAATGAAAGAAAGAGAGAGTCTTC
TTGTTGGAGTAAACCCAAACGGTTTTAGATTACTTATTAGCTGTTCATCAGTTCT
TCCTCTCTAAAAGAGTAAAACCTAAACATCTCTCTCTGTTCTATTAGAACCAAAG
ACCAATCTTTGTGAACAAAACACATCTCGTATACTTCAGATCTAGACTCGAAAAT
TTTAGACCTCTTTACAATTGGTCTTTGTTCATCTGAAGTTGGAGAAAATAGTTAG
CTTAGGTCGGATCTTTTCATATGCTTTGGATCCTCCTTCGTCTCTTTTGTATAATT
TTAACCTTATCAAGAGTTCTTTTTGAATCTCAAAAGATTATATAGTAGTATAGAAG
GTTTATATGTATATGTATAGCCAGATAGTTTATGTTGTTTAAAGATTCGATGATAG
CCAAGTTGGGTTAACTTTCTTTTTCCTTGCCTCCTTACTCACATACAAACCCTAT
CTGTCCGTACAAAATACTAAAAACCCTAACTTTTCTCTCTCCACCAATCTAGTTT
ATTGTTTCATTTCCACTTCAAC

Pair1

~~CCCAAAATTACACTTACCGCCAG~~

CCCATGTGTTCCAGTTTGTTCC

Pair2

CTCCTTTTCCTTCTGCTGAGG

TGGCGGTAAGTGTAATTTTGGG

Pair3

AAAATCCGTGTCTGCCTCTTCC

CAAAAGTAAGTTTCCTCGGTCGG

Pair4

CGTGTACGTTTAGGTGGCATG

GTGATGAATATGTGAGGTTGGGG

ABI5 promoter

AGGCTTAGACAGCCATTAATTGTGAGATGTGTCAGAACAAAATTAGGTGTATTAA
TTGAATTATGGGCCACTTGTGTTTGTGTTTGCATGGTCTAAAAACAATCAAAGGA
AAAAAGTAACTAAAGAGGCCAATATGATAATTAATAGTTAAGTTTATATAAAACC
GCATTAGGCTAATTAGCATCAATCCGACCTTAGCTCAGTTGGTAGAGCGGAAGA
CTGTAGTAGTTGCTGTAATCTTTAGGTCGCTGGTTCGATTCCGGCAGGTCGGAT
AATCTTTTTTATTTTTGTTTTTAGTTTTTTGGCTATTAGAAACACTTGATAAATTAA
GCATTTAAAATTCGATAGTCCTTATGCAGTGAATAGTCCACGTGCACTCCCAAT
GGAAGTTCGGAATCATGTTTTTTTTTTTTTGTCAAAGTTAAATAAAATATCTTCGA
AAAAAAAAGTAAAAAAGGAAGAGGATAATTTCTCGACTTTGATTTGTTTTAATTCT
TTAATAAGTTTTTGTCTCACTAAACGTAATTCTATTCAAAATCTAACAAGTCTACT
TTCACCAGCTAGAAGCTCAACGACAAACAAGTGTTTACAAACTTTGACTATTTCT
TTCATAACATCTTGAATCTAACCTAAATATGTTTTTACCAAAGTAAATCAGTATTG
ATCAATCAAATTAATCCGTCAATTTCACAAAACAAATCATGAACCAATATGTAAAA
AAAGATCACACTTTAATTCGCTCTTCTAATCCAAATTTCACGTTCTTATTATCTTA
TATATGCTTAAAAGAGAGAAAATCATGTCTAGTTTTAGACGTAAAAAAACATTTG
TGTAGCCGAAGTCACACGTGTCGAGCCTGTGAGAAGACACTGCAAGAAACAGA
AGAGAGACACGTGCAGGACACGTGTCGTCCGCAGCCGAACGGATTCTTTGTCT
CTGATCATGGCCTGGACCTGGACCTGTCTAAGTTAGCATTCCATTGGTCGAAA
GATAACTTTAGACAAAAGATGTTGACCTTCACGCCTCTCTTCTTAGACCCCACG
CGCCACACTCTCACGGTGAGAACATAAATATCAATCTCTCTTTTACCGCCTCCTA
CCCATTTATCTCTCTCTTTCTCAAAACCTTTCAGTCAAAATTCTCCGGCGGCTTT
TAAACTATGTGAAGGAGGAGAACCTCCATAACAAGAAGCGGATTCTCTCAGTTT
TCCGGCGGCGGAGGAACACAAAGCCACCGGTTTTTAGACACACAGATTTCATTT
TCAGGTTAACACTTTTCTTGTTTTATAATTTTTCAAGATCTTGGATTAGAAGACTG
TTAAATAAATCTATCTCTTTATTTATAAGTTTAGGTCTTCGTTTTCTGGTCCTTACT
TATCTGCGTATTACCAAATATTAAACTTTGTGGTAAAGTGATACTTAGTAATAGA
CGGATATAATGATGACCCAAGCTAATGATTAATTTATTTTACTAATCATCAGGTTA
AGCTCTAGATTATCTTTTGTTTAAAACCGTATTAATAAATGATTAAAGAGGAAAAA
AATCTTGTGTTGATAAGTTCGCTTTAAGAAGAAAACGAGTGGGTTAAGATATTTT
CCTCTTTTGGTAACGAAAACTTTATTGGAAGAGTAAATTATTAGTTTATTTATTGC
TAATTTTAGTATCGTTTGTCGCTGTCACGATGTGGACCGTTCTTCTTTATTTTCAC
GTGGACCGACTTAATTACCTTCTTTCATTCTTGTTATTTCAATATTTTTTACGTCA
TTTAAACTCCACTTTACCAAAATAATTTATTTCATTCTTTGAGAGCATCAATTATC
AAATAATTCAATTGTCTTTTTTCGTGATAGTTGAATAAGGGACAACTATTAAGTGA
GTTTCGTGAATAGCTGAACAGGGACAAGTAACTGAAGTTTGGTCAAAATAATAC
GATGAATTTAATATATAAGAGACAAAATTGTTATTGTCGTTTAGGTGGTATCCAT
ATTTCAATATAGTTTGTGTGAATACAGATATAAAAATGGTTATTGTTGTGTATATG
ATGCAGTTGTTAAATGGTAACTAGAGAAACGAAGTTGACGTCAGAGCGAGAAGT
AGAGTCGTCCATGGCGCAAGCGAGACATAA

Pair1

CATTTGTGTAGCCGAAGTCACAC

GCACGTGTCTCTCTTCTGTTTC

Pair2

AAACAGAAGAGAGACACGTGCA

AGGCCCATGATCAGAGACAAAG

Pair3

TCCTTATGCAGTGAATAGTCCACG

AAAACATGATTCCGAACTTCCATTGG

Pair4

CGATGTGGACCGTTCTTCTTTA

TGGTAAAGTGGAGTTTAAATGACGT

CBF3F	TGTCTCTGGTAATGCCACGTA
CBF3R	TCTCTGGACACATGGCAGAT
ABI3p1F	CGGTTGTTGACTCAATTGATACG
ABI3p1R	ACGATGGTGATGAATATGTGAGG
ABI3p2F	CGTGTACGTTTAGGTGGCATG
ABI3p2R	GTGATGAATATGTGAGGTTGGGG
ABI3p3F	CAACCTCACATATTCATCACCATCG
ABI3p3R	CGTCGGTTAGGATTTCCCCAA
ABI3p4F	AAAATCCGTGTCTGCCTCTTCC
ABI3p4R	CAAAAGTAAGTTTCCTCGGTCGG
ABI3p5F	CTCCTTTTCCTTCTGCTGAGG
ABI3p5R	TGGCGGTAAGTGTAATTTTGGG
ABI5p1F	TCCTTATGCAGTGAATAGTCCACG
ABI5p1R	AAAACATGATTCCGAACTTCCATTGG
ABI5p2F	CATTTGTGTAGCCGAAGTCACAC
ABI5p2R	~~GCACGTGTCTCTCTTCTGTTTC~~
ABI5p3F	~~AAACAGAAGAGAGACACGTGCA~~
ABI5p3R	AGGCCCATGATCAGAGACAAAG
ABI5p4F	CGATGTGGACCGTTCTTCTTTA
ABI5p4R	TGGTAAAGTGGAGTTTAAATGACGT

NCED6 promoter

TTTTTTTGTATATTTATAAAATTACAAATTAAAAAATATACATACAGATAACGAACA
ATCCGAAAAATAAAACTAGGAAAACGTGATTTTTCCGGAATTTTAGCTCTAAAAA
AGAAAACTAAACATACAACCAAAACCAATAAATAGACTAAAAACCGTATTGGATT
GAAAGCCAAAGTCTGAGACATAAAACTAAGAAAAATTCTGATACCAAACATTAGT
GACGCCTGCAAAATTCCAAACTTCTAAAAATCTAAAGAAAACAACAAATCGAAAA
AATCTAAAGAAAGCCAACACAACATAACAAATTTCTCCTATGATGAATAGAGAGT
ACCTCACAAATCAAGTGTTAGCATTGTATTTTATAGTGAGTAAGAATTCTAGCTA
AACCCAAATAAAATAAAATTGAACATCATTACTAGGTCTTTGGGAATACTCGCTC
ATTCTTCATTCATTTTCTTCCTTTGAAGCATCCTTGTCGTTTCATAGAATGTTTTA
TCTTTCTCCTTCTTAGCTCTCATAATCCATATTAGACTGTGGATTAATTCTCTAGT
ACTCTAATCTAAATTTTATCTACTATGTTTATGTTTCCGACTTTTTTTTTCACTATG
TTTCCGACTTAGATGTTAGAAAATCACCCTTTCATGTCATATCAATATTTTCTTGT
GTCTATCATTGTACACTTTAAGGCTCACTTTCGAGAGATCGATCCGACTTTTCAA
ATCAGTTCTTCATCAATAGACGTATGTCTCTTACATATAATCAATCAGATGACCC
AAAAAAAACTATTTGAAATAGTGAATAAAAGTTAAAAATTATTTCAAATACTTCAT
ACAAATATAATAAAAATAAAAATAAAATCTAATTAAATCTATAAAATTGTAGTTTTA
AGATACCATTGTATTAAATGGACATATCTCCTTATTCCGAGAGCCAGATTGAAGA
CAAATTTGTAAATGAAAATGGTCGATCAAAGATAAAATTTTAGAATGGCCACTAG
CATTAAAGTAAATTAGTGGTTTGATATATAAACTTTGTTTAGCACACTACCACCAT
CGACTTCGAATCTCCGCAATATTGCCACCACCACCTTCCAATCTCCGCATTGTT
GCATTTTTCATTATTTAATGCTGTAAAACAAAACTAAACTGTGAATCAAAATAAAG
CATACCAAAATTTTCTGACTTGGCGGCGTCTAATAACCAATCCAATCCGATTCAA
GAAACTTGGACTCTAAGCGACCCTAACAAACGGTCCACTACCATCTCCCCAGTG
TATTCCACTAGCAACTTCACATTATTCATGACACATTTAATCACAATAATTGATTA
GTAACTTTTGGGGACTAAATTTAGATGTATTATCCTTACTAGTACTAAACGTGAA
AGTTTTATCACACACCGCATGAATTTTACATTATAGTTAAAACACACACACCTGC
CACAAGGTCATACCAATTATATTACGAATATAAAAAAGTCTTTCCACTGTTCCAC
ATAATCTTCCTTCTTTATCTTCCACAATATCTCATTCACCAGCGGTTACCAAAAG
AAAAAAAAAAAACTATTCTCATTCACTATGCACATTATTGTGGCAAAACAAAATCT
AATTCACCAACGGTTACACAACTCTATGCCAAACTTTGTTTTTCGAAATTCTGTA
TTCTGAAAACATTCGACTAATTTTGTACAAAAACACATGTGTACACGGGCAAAAA
AAGCACATATCTATTATTTTAAAAAAGATTAACAGATTATCGTTCTTCCATTACTT
TGTAATGAATGACTAATTTTTTTTACTAAACTTTTATATTAAACCTGTGTCTAATTT
TATGACTAATGAATTTCTTTTGACTAATATTTAATTATTGAACACATGTGTTATACT
CATTGGCAATAAAAACACACATAGATGTCTTTAGTTAATAAAATAAATTATAAAAA
TGTTATTCAAAAGAATATTATTGGTCTTCCAATATACACTTTCTTTGGATTTACTA
TGAAACAGACAATCTTCATAAAATTTAAAGAAATCTATTAAAAACAAAACTTCAAA
TTACAGCAGACCCTATAATAATTGTAAGGATTCACTTCTATATATGTGCCTGATT
AATAAAAGGTAAAAAATCTTATAGTTTTCTAAATTTATAATTACTACTTTTGGGAG
ATTTGATACCTATTTAGAATTCACTCTCAAACAAAAAATTCCCACCGTTGAAACA
AAAAGTAAATGTGTGTGAACCAATCAAAATCGTTGAATTAAGGAAAAATCTCCAC
ATTATAGCATACCTTATAATCTGGATATTTTCTGCTATATATGTCTCTAATAAAGT
AAAATTGTAAAAAGAACAAAATATTACAGTTGTTATTCCTATACGTATACGTAATA
ACTATTTTTGGAAGATAAGATTCGACACCTATTTAGGTTCACTTTTAAACCAAAAA
AAATCTCTACCCTTGAAACAAAGAATAAATATAAGTAAAACAATCAGAATCGTTG

GATTAAAATAAATCTACAAAATACTCCACATTATAGTAGAGAACATATAATCTAGA
TTTGCTATTAAATATGGTATCTTGACAAATGTCAAGACCCCGTTACATTTTCTATC
ATCTTTATATAAACCGCTAGCTTTTACCCTCTTGTTTTGGGCAAACACCAAAAAC
GTTTTTACTTATAAAACGTTAGAAAACTTCTCTAGTCATGTTTTATGCATAGGTCG
CTCACAAGTCACCAC

Pair1

ATTCTGTATTCTGAAAACATTCGAC

TTTTGCCCGTGTACACATGTG

Pair2

GAACACATGTGTTATACTCATTGG

GAAAGTGTATATTGGAAGACCAATAAT

NCED9 promoter

TAAACTTTTTATTTTGTTAAAGGCTTAATTTATAAACTTTTTTTGGATGTCAATGTT
AAGTAATTTTTTTTATATAAACATACATTATGGCAATCAAAGTTCATATAGTTTAG
TTATTGCTAAATATACAATATTAAAGCCTCACATAACTATATAATTTTGGGTACTA
CATATTAAGTGTTTCATAAAAAAGATTTATCAGGTTTAATTAAGTAAACAAATAAC
AAGCAACGACCATGTTACTCATAATAATGGTCTTGCGAGCCAATCATATAGTTTT
TTGTTTGATAACCACGCCAATCATAAAGTTTGTTAGTTACCAGTTGGATTAATTA
ATGTAGATTGCTGGACAGACCGAATCCTACCTTTCAATGGATTTCAGTAAATCCA
AAGCCACTATTCATCTACAAACCAAAAATTTTAAGTTATTATATTTGTTTCTTATTT
ATATATGTTCATTTTTGCCAATAGACAATAATAGGTATATATGAATCTTGGGGAA
AAGGTGACTTGGTTCAACCATGTGCTTCCATGTGGTCACATGAACTGAACCATA
TTGCATATATATGTACTTATCATATTGTAACTACATACGTTTAATGCGCATATATG
CATATATTATATAGTTCAAACTTCAAAACGTCTGATTAGAGAAAATAATATATGAT
TATCTATGTATATTTTTCAAAGTATAGCCATAAAGAACAATAAAATTTGGTGCTCG
AGGTAGTCAAATTACAATTAGTCGAATCACTTTTATGTAGACGCATCTCTAAACA
GAAGTTAATTTCAATTCAAACATGAAATCCATTTTTTGTATTATTGTGCGTGTGTG
TTAAAAGATCCATACTATTTGTTAGCGATCGAGCAGGTCTTCGGTCTCATACATT
TGATCATAATTTTAGTTCATGTCCAAAATTGTATACCAACGACGTTGCGAGAAAT
TGGTCAACAATATATACAACTCCAAGGATGTTAATTAAACGATCCACTTAGATAA
TTAAACCATATAATAATTTTTCTTCCTGAAAATCGCAAATCCAGTATTTTATATCA
AACATTTATATCGCCAATGCCCATGGGGAGTTTGGTTTACAAATCTGAAGCTCT
GAAATGGACACCTGAAGCCTGGATGTTCCGTTAATTAAGAATATATTAAAATGAT
TAATGTAATTAGTGGACTGTGATGCATCTACGATTGCTTCATTTCAATTTTCAAG
TGGGTGTTTACTCAACGATAATGGTCATCACCAACCAACTTAAGTTAATCACTCA
ACCTAATTTTGTATAACCGAATCTTTTTTCTTTTCTTCTTCTTAACCGAATGGTCC
GAATCTCTTGTTAGAATATTTCATGTATACTATAAGACTTTATTCTGCATAACCCA
ATATACACAATTTTTTAATCCCAATTTCTCTAAATAACATAATACAAAACTTCATTG
GAAAATTTACGATAGCAATTTACTGACCACGACAGATTTGTAGTGATGTATGAAT
AATAGAAGCCGTAGACACAAAACTATAACAAACATCTCGAGGACCTAAAAAAAA
AGAAGAAGTGAAATTACTAAGTACTGTAGCAAGTGTATGTTCTAAAGTTCCAATG
AGTAAAATGGCTCAAACAACCGATTCATAAAATATATTTTCAAAAATGAAACATTT
ATATATACCCATGGTGTTTAACAAATTCGTAAAATGTCATACATTTTCGGTACAC
AAATAATTTTATAATCTCACTTACGCGTTGAAACAAATAAGCAAGCGCGTAAGAC
ATACATTACACTTTTTTAAAATGACTGGAATGCATGACGTTTGGGCACATTAATT
ATGTAATTTGTTCGGTGCTTGTGTTGTGTGTATTCTTCGTCAGTGAGAACGCAAA
AGTTACGAACCAATTTATGGTTTCAAGTCAAAGACAATTGAAAAGACAGTTTATG
GTTTACGT~~ACGTGGTGGTTCGATGTAAAACG~~TTTTGATGTGGAGGCCACAACGC
GTCTTAACCAAAAAGCCAATTGTCACTTCCATTACCAGTGGAATACACCAAACCT
GGCCTATTCATATGTTTATATGCATGCCCATGTTTTTTAATGTCAAATCTCTAAAT
TCTAGTTGGATTTAAGTTTCACGTACTTACGTAGAAGCAGACGAACTTCGGGGC
AGTACTTAAATACTATTATCAGACTTAATTTTGAAAAGGAATTTTTTTTCCGGATC
AACATCGAACTAAAGGTATATGATTAGAATCTAAACCAACTACATCCAGGTTCAG
TCGTCCGACTAATTTTAGTACTGCATCAACCCGAGAAAAAAAGTATAGGTTATCG
AATTTTCAACTAGTCTCAGGTACCAAACTATAAGGATCTGGTTCAACGTACTTTA
CAAGAATTAGACCACTCAATAATACCGACAGTCTCAGTCTCTCTAACACGCATG
TAATTTTATTATTTAATGAGATTTGAATCCAAGTAAAATAATTTACACATTTCTCAA

AATCCAACTATTACTACGACCACAAGTAATTTGGACTATTAGGTATAAAAATCAC
TTAAAAACTAAGACTAATAATTGAATAAGTTCCCATAACGAATGTCTCACATCGT
TGGTATCATCTCAATTGTTAAATTTGTTTGTTTTATGTTACGTGAAACTACTAATA
ACTGCACTTTCCGAAAGAAGACAAAACAGTTGATCTCAAATATTTTGTTACCGTC
CAAGAGGTAGTTCTTCCATCCTTCAATGACATTGTAAGCACTTCTAAAACAAAAT
CTGATCAAAACTACTTCAAACACTTCTAATACACAAAAATTGATCTCTTAAACTAG
AATGCCGGAAAAAAATATATCTCTTAAAGTAGAAAAATTTCTAAGTGAAATGATC
AAATTTATTACAACTTATTATCCAAAGATAAATTGTGGGAGATAAACAAGATGAG
CCATTTTATTGAAAATGATAAAGAGGGACGATGAAAATATGTAAGTCTCGTCAGA
TAATTATTAGGACACGTGTTAAAAGATGAGAAAATAGTAGCGTGA

Pair1

CTTCGTCAGTGAGAACGCAAAAG

AACGTTTTACATCGAACCACCAC

Pair2

ACGTGGTGGTTCGATGTAAAACG

ATGGAAGTGACAATTGGCTTTTTG

Pair3

GTGGGAGATAAACAAGATGAGCC

TCTCATCTTTTAACACGTGTCCTA

CYP707A1 promoter

ATATATATACACACTGATACATTTATATATCGCATATATATGTTTGTAATCTTGTAT
ATATATCTAACGTATGGATGCATGAATTTTACAAAATGAGAGAACCATTTATTGG
TAAGACTTTTCTTTGATATCATGAAGGAAGCAGCCAAAGTAATATATATTTTATGT
TCTGATTATTATATTTTTCTCCGCTCCGCCAAGTCAATTGATAAATTTCGTGCAA
CTAAGAAAAATATCTGCATCTTAAGCTAAGTCATATCATTAGAAATATAAATATAG
AAGTCATATTCTTATGTTCCTTTTCTCTAATCAGATTACATTTAAAACCCAAAAGT
TTGAAGATATATACTAGAGTTATTTGGCTTTGTTACGTTAACGTGGGAGGAAAAT
AGCGAGTGTTTCTGATTAAAGTCAAGAGCAAATGAGCAATACATATTTGTCTGTA
TATGTATCCATGAGTTACTAGTCGAAAAAAAAATGTTCATTACTTTTATGTTATGT
TTATTTTTGTCAACAGGTCGTTGCAGATTTGCAGCTTATCTTAACCCTAATATTTT
ACACATATGTCATGTATGTATTTCTCAGAATCAAGTTAAGAGGTTAAACAAAAAA
GGAGGTTAACCAGGGTTTGTAGCTTATAATATATTTTATATTTATTATATGTAGTT
TATATTAATTTTTGTATATATAAGTGAAAAAGCTATGTAACAATATTGTAAAAAAC
AACATATCATTTAAATTATAGCTCCATAATATATATAGGGAATAGAAACAACTCAA
ATGTAAATAATATAGTGGTAAGACTCCTAAATTATATAATCCTTAGGCCTCAAAA
AAGAATGCGTTAGAATGTAAATTAACGGTTTAAAATTTTCTGAGAAAAAAGATGA
TGAGGATTCGGTCCACAATTCTCCGATTAACCATATAAAACTCTAAACTTTCATA
TAACTCTTATCATGAAACCGTAAAAGATCGTCCATAATTATTGATCCAGATTTTTT
CATTAAATAATAGAAGGATTCAATATGATTTTAGCTAGATAGATTTGGGAGTTTA
ATTAGCTGGTTTAGCTAATTCACCAGAAAATGTTTTATCTAACTAGTACTAATTAG
CATATTGTATTTTACATGTTATCCCAAATGAATTATTTTTAATTTTCACACACACA
CACATGTATACACTTTTCATCTAGAATGCCACACATCTTCATTTTACTTTTAGTCA
TTGTTAGTTGTATTTTTTTGTTAATGAATGATTGAAAACTGAAATTGCAATATAAG
AATATTTTGATAAAAAAATATATATGACACAAATGCAAATAATAATAGAGAATTCT
CAATATAGAATTGTTTAAAGAAATCTATCATATTAATCTAAAAAATTATTATCAAG
ATATAAATTAAAAAAAAATATCTTATCTAATTCTATATGTGCATGTGGTATGTGTA
ATAAGCTACTACAGACACGTAAACAGATATTACTACTTTGAATAGCAATTACATT
TATAACTTTACAAAGAAAGACTACATATGTAGTAAATAAATCTCAATATTCAATCT
CGTTTAGCCATTTGTCATGATATTTGTATGTAGATATGCTATATAATATCACAAAT
ATCATATTCAAGTAGTAAAAAATCTAAAGAAAAAATAGTTGCCAAAACAAAAGAG
ATAAGTAAAACTAGTCTTCGTAGTTATCTCTCTGAATTATGCATGGATATCATTC
CCACTCTTTTATTCACTCTACTATTAAGAAAAAAAAACGTGCTGTATATGAAACAA
AAAAAAAACCCAAAGAAATTGTTGATGTCAATTGATATAACACATATCTGAAATTA
AATATTTGATGTAGCACAAGAAACTTAAATTTAAAAACCAAATAAACTTATAAAGG
GTAGACTTTACCCCACATTTGTCTCTCTCTACTTAAGCCAAATTGCTCTATTCTTT
TGTTTTCTCTTTATAATTAGGCTTTATAAACCAACTTTTATTAAGAACAACAAAAA
TAAAATTGTTATTTATTTGGCAACAGAACGAATTATTAACCAAATTAATCCATTTG
AATGGCCCTCCTCTCTCTCTGTCTTTAATTAGTTGGTTTGGTCAAAATCTCACAC
GCTCTTTATGCAT~~GCTCTTTGACACGTGCTATCTTCG~~TCTCTCTCAAATGAGCTG
TCTCTCTAATAAGAGTTTCTTCTGTATAGGAAAGGAAAGGTCTCTCTCTCTCTAT
TTACTTATGAGAATAGCCTATAAAACCCCTTCACATCTCCCACTTGTTTTGCATC
ACAAACATTTCTTCCATTACATTAAAACTCCAAAAAATTCATTTTTGTTTTCTTTTA
GAGTTCACAAGTTCTTCGTTGTTCAGCTACTCCCACTGTCATAACACGAAGTGG
GTTTTTTTCTGATCAAGAACAAAAACAAAA

Pair1

ATTTGAATGGCCCTCCTCTCTC

AGATAGCACGTGTCAAAGAGCA

Pair2

GCTCTTTGACACGTGCTATCTTC

GAGAGAGAGACCTTTCCTTTCCT

Pair3

ACTTTACCCCACATTTGTCTCTC

GAAAACAAAAGAATAGAGCAATTTGGC

Pair4

GATTCGGTCCACAATTCTCCG

TGGACGATCTTTTACGGTTTCATGAT

CYP707A2 promoter

GAACAATAGAGATAGATGTTTTACAAAACTACATTAAGAAGTTATAGATGATTATT
TACCAAAAAAAAATTATAGATTCTTCCATGAAAATTAATACGATTTTATTTATTAAA
TTGTTATTATAGATTTATATAGAATTCATCTTTCAGTGCTTGCCTACTCAATATTT
GAAAGAAGATACATCAAACGCACTTCTATTAATTAAAGTTTGTGAAGCAAGTAAC
ATCAGAACAGAGATGTCTACAAATAAAAGTAAGAACATTTATATTCTTATAACAAT
ATACATAAGACTTAATATCAAACCCAAAAAGAAACAGAAAAAGAAAAAAGACTGA
CGACGATAACTTAAGAAAATTTCATCATTATGCGATCACCGACCAAAGATGAATA
GATGATTATACGAATAATATTAAGACTTTTAAAAAACGAAGGGAAGTTTCTTAAG
GAAGAGGAAACGTTGAAGTGTGAATAACTGAGGAGTTCCAGCGAAGCGGCTAC
TGCATTTTGCAGTGATCTGAGGTTCGTATATGCGTACGTGGATTTCTAGGGATG
TCACGGACTAAGTTATTTTTATTTTAATCTTCAAATGTCATTTGATTTATATTTTAA
AGATCCTTCTCCAACTTATCCACTACGCAGCCCTTTCACCACAACGAACGACCA
TATACGTCAATTTTAAGTGATTCATTGACTAAAGATAACGTTAGTTTAAAGTATTT
AATTTACCAAGGTCCAAGATATGATGAAGTTTTCAAAAATAGTTTAGAAAATAAG
GTTTTTGGTGACCAGAGTAAATTTTATTTAAAACCTTGCATTTTATAAAGGTAAAA
GATCGTCAAATATTCTTTTTAATCCATTCGTTATAGAAACTATTAATTATTTAATC
GTTTTCTTAATCATCATGTTATCAATTGTTTTCTATTGCAAAGATTTTTTGTATTAT
CTTCTATCGTCATTGTGTATACACAAAATACGTAAAAGTAATTGACTATCACATC
GTACAGAGAAAAGATTCATCATCATAAAACAAAAATGGAAAAACTCAAACGTAGT
ATTTGTTTAACTAATGGAAGTGGTAATATTTTCTAAACAAAAGAAAAGAATTTAAC
TTTGCTAGATTTTTTCTTGCTGACAAGAGAACTCAACCATACAACATTTTCAGTG
GATCCCATTTATCTCAAAGAAAAAATGATGAACTAAGGTCTTTGTTTTTTGTGGT
CACTAAAGTGTAGTGTGGGGTTAGCCAAAGGGCAAGTAAAATACAGTCCAAATA
CACAAACACCACTCACCACTGAATTTACACATTATTTTTCCTTTCTTGTTCCGTAC
AGGTTTTCGTATCAACAAAAAATATAAATTATCCTAAAATAATTAATCGTAGAACA
ACAAAGGATAGCAAACGATAACCACATAAATAGTACTGCGCCGAACACTTTGTG
TACAAATATCTATATATGTAAAGACTCAAAAGAAAAGTAAAATCAAGTTTTGAAAG
GAGAACATGTAAATAACATTCAAAAAAAAAAAAAAAAACAAGTAGAATGATAAGTT
CTGAATTATACTTTTAATATACTTCTAGAATCTAGGATATTCTATTTGTAGTATATA
TGCAATTTTCAAGTTTGTGTTTATTGGACCAAAGAACCGTCGGCAAATTTTTGTA
TTACTTATCAAAAATATATTTTATTTTAATTACTATAAAAATAAATACAAAAAACTA
AGATAGATATACGATCTTTATTTTTTCTTTTTGCATATATTAGCTATTCGTTGGCG
GCAAAATTCTAATTAATATGTATATAATTAATTACAATAAATAATAAATGTTTGAAC
AAAAAAGAAAAAAAAAAAGAAAGAAAGAAAGGCCAGGAATGAAGTATCTTTCCAT
CTCAACTATAGCTATATAAACCCCTCAATACTTCAAGCAAAAGTCACTAACAAGA
ACAAACAAACACACACAACTCATAACTATAACTATACATTCATACATATAACAATA
ATTCACTTAAATCACTCGGAATAATGCAAATCTCATCTTCATCGTCTTCAAATTTC
TTCTCTTCTCTTTATGCTGATGAACCGGCACTAATCACATTAACAAT

Pair1

GCGTACGTGGATTTCTAGGGATG

GCTGCGTAGTGGATAAGTTGGA
