# Supplementary material for: ICE1 and ZOU determine the depth of primary seed dormancy in Arabidopsis independently of their role in endosperm development
Source: Plant J. 2019 Feb 18;98(2):277–90. doi: 10.1111/tpj.14211 (PMC6900779; doi:10.1111/tpj.14211)
Supplement: Supplementary file 7 — Table S2. Testing the significance of the ice1‐2 and zou‐4 dormancy phenotypes over multiple experiments. [file TPJ-98-277-s007.docx]

Supplemental Table 2: Testing the significance of the *ice1-2* and *zou*-4 dormancy phenotypes over multiple experiments. 2-way ANOVA to show the significance of the seed dormancy effects of loss of ICE1 (n = 30) or ZOU (n = 20) over multiple experimental repeats. D.F.: degrees of freedom. V.R. variance ratio, between treatments vs within treatments. P: probability of accepting null hypothesis by chance. Statistical analysis was conducted in Genstat version 18.1 (VSN International Ltd).

| comparison | **Col-0 vs *ice1-2*** | | | | | | | | | | | | **Col-0 vs *zou*** | | |
| --- | --- | --- | --- | --- | --- | --- | --- | --- | --- | --- | --- | --- | --- | --- | --- |
| Germination test | **No cold** | | | **1 day cold** | | | **3 days cold** | | | **7 days cold** | | | **No cold** | | |
|  | **D.F.** | **V.R.** | **P** | **D.F.** | **V.R.** | **P** | **D.F.** | **V.R.** | **P** | **D.F.** | **V.R.** | **P** | **D.F.** | **V.R.** | **P** |
| Genotype effect | 1 | 530 | <0.001 | 1 | 646 | <0.001 | 1 | 171 | <0.001 | 1 | 95 | <0.001 | 1 | 659 | <0.001 |
| Experiment effect | 5 | 2.9 | 0.023 | 5 | 2.5 | 0.039 | 5 | 13 | <0.001 | 5 | 23 | <0.001 | 3 | 2.2 | 0.103 |
| Genotype x experiment interaction | 5 | 1.9 | 0.104 | 5 | 3.8 | 0.005 | 5 | 15 | <0.001 | 5 | 24 | <0.001 | 3 | 0.06 | 0.062 |
| Mean genotype effect size relative to Col-0 | -83.9% | | | -77.1% | | | -34.8% | | | -17.4% | | | -81.6% | | |
