# Supplementary material for: ICE1 and ZOU determine the depth of primary seed dormancy in Arabidopsis independently of their role in endosperm development
Source: Plant J. 2019 Feb 18;98(2):277–90. doi: 10.1111/tpj.14211 (PMC6900779; doi:10.1111/tpj.14211)
Supplement: Supplementary file 8 [file TPJ-98-277-s008.docx]

# Legends for Supplemental Tables and Figures

Supplemental Figure 1: The dormancy phenotypes of *ice1* and *zou* are repeatable and robust. Data are averages of five biological replicate seed batches with at least 25 seeds per batch ± SE. These data are from separate experiments some of which are presented elsewhere in the paper. Exp1 = Col versus ice1 alleles, Exp2 = GFP rescue, Exp3 = Double ice1/zou, Exp4 = Excision of embryo, Exp5 = Double aba2/ice1, Exp6 = Double dog1/ice1, Exp7 = After Ripening, Exp8 = Exogenous GA, Exp9 = Exogenous Norflurazon

Supplemental Figure 2: The increased dormancy of *ice1* or *zou* can be rescued by exogenous gibberellin (GA_3_) in a concentration dependent manner or by after-ripening. **A.** The germination frequency of freshly harvested seeds of wild type (Col-0), *ice1-2* or *zou-4* sown on media supplemented with exogenous gibberellin (GA_3_). Data are averages of five biological replicate seed batches with at least 20 seeds per batch ± SE. **B**. The germination frequency of freshly harvested seeds, or seeds that had been after-ripened at room temperature in sealed tubes for the time indicated of wild type (Col-0), *ice1-2* or *zou-3*. Data are averages of five biological replicate seed batches with at least 25 seeds per batch ± SE.

Supplemental Figure 3: ICE1-GFP is located in the nuclei of stomata of true leaves as well as endosperm of developing seeds. Representative figures are shown and white scale bars indicate 50 µm. Auto-fluorescence is in purple while GFP in green. **A & B**. Images of the abaxial side of wild type (Col, A) and ice1-2 pICE1:ICE1-GFP (B) true leaves from flowering plants. **C & D**. Chloral hydrate cleared heart stage developing seeds of (Col, C) and ice1-2 pICE1:ICE1-GFP (D). Equivalently staged seeds were used in E – H. **E & F.** Maximal projections from Z stacks collected at 0.5 micron intervals in developing wild type (Col, E) and ice1-2 pICE1:ICE1-GFP (F) seeds at heart stage. **G**. Close-up of F. **H**. Reconstructed plane of a ice1-2 pICE1:ICE1-GFP with coloration demarcating external (red) to internal (purple). Image is created from auto-fluorescence as well as GFP signal.

Supplemental File 1: putative ICE1 binding sites in the targets in figure 5. Word document showing the locations of the putative ICE1 binding sites in the promoters of the genes used in Figures 5 and 6.

Supplemental Figure 4: Chromatin immunoprecipitation (ChIP) using endosperm-enriched fractions of *ice1-2* *pICE1:ICE1-GFP* shows no evidence for enrichment at putative ICE1 binding sites in the promoters of CYP707A2, CYP707A1, NCED6, or NCED9. Data represents the average ± SE of three biological replicates per locus. Primers in the 3’UTR of ACTIN2 from Adams et al. (2015) were used as a negative control.

Supplemental Table 1: Sequences of all primers used herein and where appropriate their references.

Supplemental Table 2: Testing the significance of the *ice1-2* and *zou*-4 dormancy phenotypes over multiple experiments. 2-way ANOVA to show the significance of the seed dormancy effects of loss of ICE1 (n = 30) or ZOU (n = 20) over multiple experimental repeats. D.F.: degrees of freedom. V.R. variance ratio, between treatments vs within treatments. P: probability of accepting null hypothesis by chance. Statistical analysis was conducted in Genstat version 18.1 (VSN International Ltd).
